# EPHA2 and WNT reciprocally regulate MAPK-dependent gene expression in a pancreatic cancer model

**DOI:** 10.1172/jci.insight.202630

**Published:** 2026-09-08

**Authors:** Shawn R. Wadia, Changyuan Hu, Siddhi Patnaik, Shreya Sridharan, Roger J. Daly, David M. Virshup, Babita Madan

**Affiliations:** 1 Program in Cancer and Stem Cell Biology, Duke-NUS Medical School, Singapore.; 2 Cancer Program and Department of Biochemistry and Molecular Biology, Biomedicine Discovery Institute, Monash University, Clayton, Victoria, Australia.; 3 Wenzhou Medical University-Monash BDI Alliance in Clinical and Experimental Biomedicine, Wenzhou Medical University, Wenzhou, China.; 4 Department of Pediatrics, Duke University School of Medicine, Durham, North Carolina, USA.

**Keywords:** Cell biology, Oncology, Cancer, Protein kinases, Signal transduction

## Abstract

Wnt signaling drives tumorigenesis in multiple cancers, in part through complex interactions with other oncogenic pathways including the MAPK cascade. In Wnt-addicted cancers, pharmacological and genetic inhibition of Wnt signaling activates multiple RTKs, increases ERK phosphorylation, and induces MAPK target gene expression, but the specific RTKs responsible for this MAPK hyperactivation are not known. Here, we performed phosphotyrosine-targeted mass spectrometry, which revealed robust phosphorylation of EPHA2 and EGFR upon Wnt inhibition. Unexpectedly, we found that in xenografts, EPHA2 suppresses EGFR and ERK activation. Most notably, the increased ERK phosphorylation observed in *EPHA2*-KO tumors is transcriptionally inert, as there is no concomitant increase in MAPK target gene expression until concomitant Wnt inhibition. This suggests a Wnt-activated transcriptional repressor such as GATA3 that gates MAPK signaling in Wnt-high cancers. Although Wnt-high KRAS-mutant cancers are resistant to erlotinib alone, adding a Wnt inhibitor mitigates this resistance. Additionally, loss of EPHA2 enhances these cancers’ sensitivity to both erlotinib and Wnt inhibitors. Our studies therefore identify therapeutic vulnerabilities in Wnt-high tumors, even within traditionally EGFR inhibitor-resistant, RAS-mutant contexts.

## Abstract 2

Wnt signaling modulates cancer growth by multiple mechanisms. Here we find that EPH2A, which senses the tumor environment, cooperates with Wnt to inhibit MAPK signaling.

## Graphical abstract

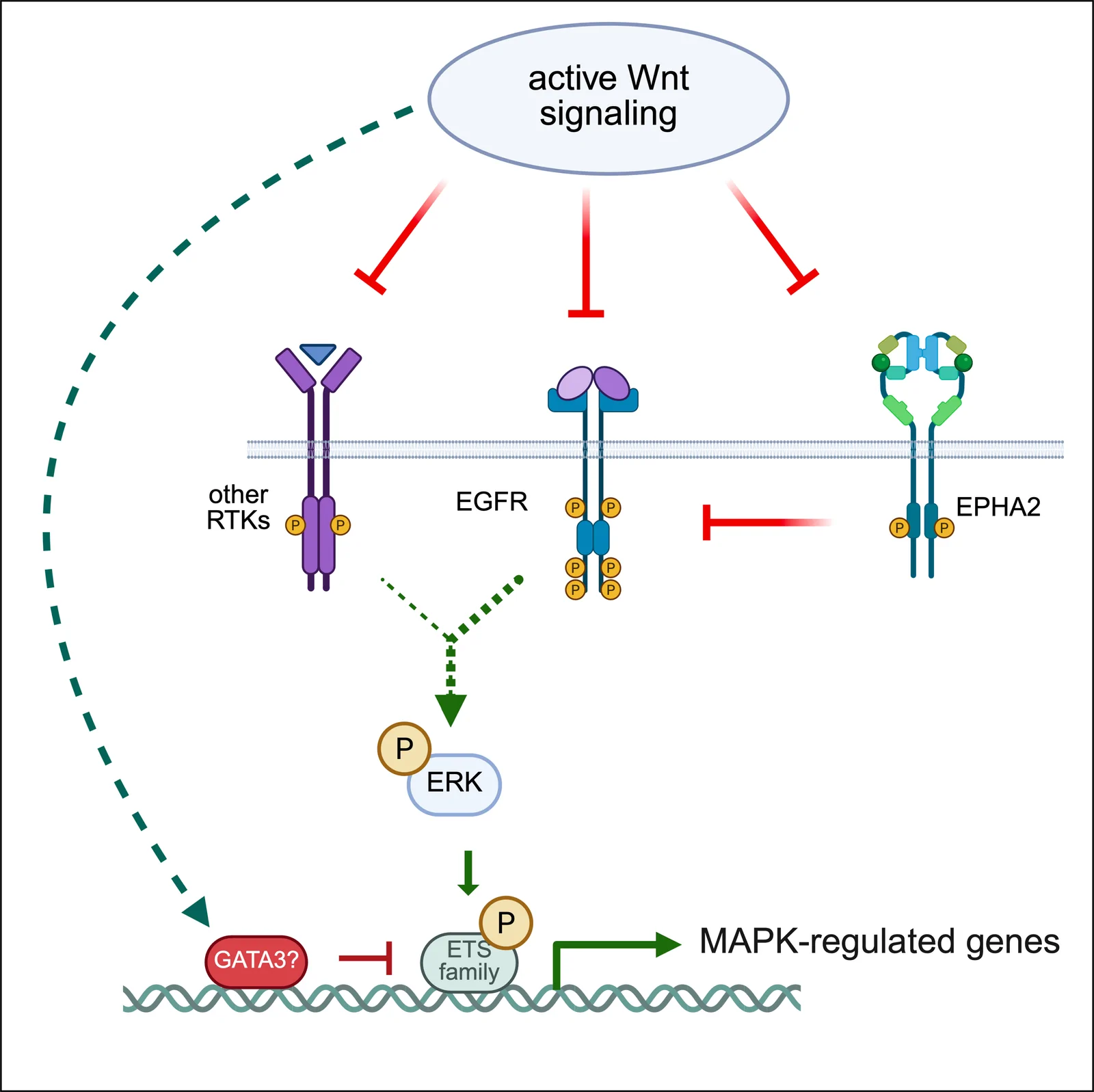

## Introduction

The Wnt signaling pathways play important roles in development, growth, and differentiation (1, 2). These pathways have 2 main divisions, a canonical branch that acts via β-catenin, and a noncanonical one that operates independently of β-catenin (2–5). Hyperactive Wnt signaling contributes to cancer development and progression (2). This oncogenic activity appears to arise primarily from aberrant stabilization of β-catenin and its downstream transcriptional effects (6). Consequently, the canonical pathway has emerged as an attractive target for therapeutic intervention.

A class of highly effective Wnt inhibitors, the PORCN inhibitors that block the secretion of all Wnts (7–9), has provided multiple insights into Wnt-regulated pathways (10–16). A key finding from these studies is that Wnt signaling represses as many genes as it activates. A subset of these Wnt-repressed genes in Wnt-high pancreatic and colorectal xenograft models are regulated by MAPK signaling (11). Notably, these effects occur in the presence of activating KRAS mutations, demonstrating that Wnt inhibition can enhance MAPK signaling beyond levels caused by constitutive RAS activation. Mechanistically, we found that pharmacological Wnt inhibition–driven changes are regulated by a C4 methyl sterol oxidase, FAXDC2, that enhances MAPK activation in part by increasing RTK recycling to the plasma membrane. However, the specific RTKs responsible for activation of MAPK in this setting have not yet been determined (12).

There are 20 RTK subfamilies comprising 58 distinct members, including 2 major families, the EGF receptor (ERBB) and ephrin receptor (EPH) family. EPH receptors constitute the largest RTK family and are subdivided into EPHA (9 members) and EPHB (5 members) that typically signal through transmembrane or membrane-bound ephrin ligands in a bidirectional fashion (17, 18). Forward signaling occurs through EPH receptors, and reverse signaling is mediated by ephrin B ligands. These interactions regulate cell adhesion, migration, and tissue architecture, with aberrant signaling implicated in cancer progression (19, 20). EPHA2 plays a particularly important and nuanced role in tumor biology. It can exert pro-oncogenic effects through ligand-independent phosphorylation at serine 897 but acts in a tumor-suppressive manner when activated by ligand binding (21). Reflecting this complexity, previous work has shown that EPHA2 loss facilitates early tumorigenesis by preventing competitive cell elimination in KRAS-mutant pancreatic cancer (22). Conversely, EPHA2 overexpression has been linked to resistance against EGFR and HER2 inhibitors in lung and breast cancers (23, 24). Although EPHA2 has been reported to both activate and suppress ERK phosphorylation depending on context, the transcriptional impact of EPHA2-mediated ERK phosphorylation remains poorly characterized (25–27).

EGFR is a well-recognized regulator of multiple signaling pathways, most notably the MAPK signaling cascade (28). Upon ligand stimulation, EGFR activates RAS, which triggers a phosphorylation cascade involving RAF, MEK1/2, and ERK1/2. Activated ERK controls broad transcriptional programs that regulate cell proliferation, differentiation, and survival. Although RAS and BRAF mutations that drive constitutive MAPK activation are protumorigenic, sustained MAPK signaling can drive differentiation, and unchecked RAS and MAPK signaling can also result in oncogene-induced senescence, making tight regulation essential (29–31).

Here, we report that both EGFR and EPHA2 are highly phosphorylated in response to Wnt inhibition. Investigating how EPHA2 and EGFR contribute to ERK activation after Wnt pathway suppression, we found that *EPHA2* KO significantly increased ERK phosphorylation through activation of EGFR. Surprisingly, however, this EGFR-MAPK activation does not induce bona fide MAPK target genes unless Wnt signaling is concomitantly inhibited. This suggests that ERK activation can be decoupled from transcriptional output, in this context due to Wnt-dependent transcriptional repressors that inhibit MAPK target gene expression. We also found that *EPHA2*-KO tumors are more sensitive to the EGFR inhibitor erlotinib. Collectively, these findings demonstrate EGFR as a critical node connecting Wnt inhibition and EPHA2 loss to MAPK activation.

## Results

### Pharmacological and genetic inhibition of Wnt signaling in vivo triggers ERK phosphorylation and upregulation of MAPK target genes.

We have previously shown that inhibition of Wnt signaling using PORCN or tankyrase inhibitors activates the MAPK pathway in Wnt-high, Ras-mutant xenograft tumors (11) (Figure 1A). To determine whether this effect extends beyond pharmacological perturbation, we also examined a genetic model of Wnt pathway suppression in which *TCF7L2*, the gene encoding the key Wnt effector TCF4, was deleted in HCT116 and HT29 colorectal cancer cells (32). These cells harbor genetic alterations that lead to constitutive activation of both MAPK and Wnt pathways. HCT116 cells harbor KRAS G13D and β-catenin p.Ser45del, whereas HT29 cells carry BRAF V600E and truncating APC mutations (p.Glu853Ter and p.Thr1556Asnfs*3). Tumor xenografts derived from these *TCF7L2*-KO cells also exhibited elevated ERK phosphorylation (Figure 1B), reinforcing the conclusion that both short-term and long-term Wnt pathway inhibition drives MAPK activation in in vivo models.

The transcriptional consequences of this Wnt inhibition–induced ERK activation were further characterized. We curated a set of bona fide MAPK target genes based on their expression profiles in HPAF-II pancreatic xenograft tumors treated with ETC-159 and MEK inhibitor (trametinib) (11). HPAF-II cells have an activating *KRAS* G12D mutation together with *RNF43* inactivating mutations (p.Glu174Ter), leading to simultaneous activation of the MAPK and Wnt pathways. Genes were designated as MAPK targets if their upregulation in response to ETC-159 was more than 50% attenuated by cotreatment with trametinib (Figure 1C), indicating that their induction was ERK dependent. These MAPK target genes, which are robustly upregulated in HPAF-II tumors as early as 56 hours after initiation of Wnt inhibition, remained elevated for up to 7 days in the presence of continued inhibition (Figure 1, D and E, and Supplemental Figure 1; supplemental material available online with this article; https://doi.org/10.1172/jci.insight.202630DS1). Notably, the Wnt inhibition–induced transcriptional activation of MAPK target genes in the pancreatic cancer model was also observed in 2 colorectal cancer models. Reanalysis of published datasets showed that *TCF7L2* KO in HT29 and HCT116 colon cancer cells similarly induced many of these MAPK target genes (32) (Figure 1, F and G). Moreover, reanalysis of data from a separate study showed that TCF7L2 knockdown by siRNA in MCF7 breast cancer cells produced a comparable upregulation of MAPK target genes (33) (Figure 1H). Taken together, these findings corroborate and expand upon the finding that inhibition of Wnt/β-catenin signaling by either pharmacological or genetic means leads both to ERK activation and a conserved upregulation of MAPK target genes across multiple tumor models.

### Wnt inhibition induces phosphorylation of specific RTKs.

Wnt inhibition with PORCN inhibitors in Wnt-high HPAF-II orthotopic xenografts leads to widespread RTK tyrosine phosphorylation, providing a potential link between Wnt inhibition and ERK phosphorylation (Figure 2A) (12). To confirm and extend our previous studies, we probed lysates from vehicle- or ETC-159–treated mice bearing HPAF-II orthotopic tumors and WT or *TCF7L2-*KO HCT116 tumors using phosphotyrosine-specific antibodies. We observed a robust increase in tyrosine phosphorylation, particularly of proteins in the approximately 140 kDa range in both HPAF-II and HCT116 xenografts (Figure 2, A and B). Further demonstrating that this is a general effect, Wnt-driven MMTV-Wnt1 mammary tumor allografts treated with low doses of C59, a structurally distinct PORCN inhibitor, also showed increased tyrosine phosphorylation (Supplemental Figure 2A).

To evaluate the relative contributions of specific RTKs to ERK activation and MAPK target gene induction after Wnt inhibition, we performed IP-mass spectrometry (IP-MS) analyses on HPAF-II tumor lysates from vehicle- and ETC-159–treated (8 hours and 56 hours) mice, enriching for tyrosine-phosphorylated peptides using phosphotyrosine-specific antibodies (Supplemental Figure 2B). Peptides were quantified using label-free quantification based on MS1 intensity. A parallel IP-MS analysis was performed on lysates from WT and *TCF7L2*-KO HCT116 tumors. The HPAF-II IP-MS dataset identified 423 unique tyrosine-phosphorylated proteins, including 35 sites on 14 RTKs in tumors treated for 8 and 56 hours. Among these, EPHA2, EGFR, and ERBB2 showed phosphorylation at multiple sites in response to Wnt inhibition (Supplemental Table 1, Figure 2C, and Supplemental Figure 2C). Independent analysis of the HCT116 dataset revealed similar tyrosine phosphorylation of EPHA2 and ERBB2 (Supplemental Table 2 and Supplemental Figure 2D).

Western blotting was used to validate RTK phosphorylation events identified by IP-MS after Wnt inhibition. Because our anti-phosphotyrosine immunoblots consistently revealed a prominent signal at approximately 140 kDa, we focused on the EPH family of RTKs, several members of which migrate at this molecular weight. Our RNA-Seq analysis indicated that among the EPH receptor family, EPHA2 and EPHB6 were the most highly expressed (Figure 2D), suggesting their potential involvement in this signaling axis (16). Since EPHB6 is kinase deficient (19), we focused on EPHA2. Phosphorylation of EPHA2 Y588, a juxtamembrane residue identified in the IP-MS dataset, was markedly increased as early as 8 hours after the administration of ETC-159 to HPAF-II tumor–bearing mice (Figure 2E). *TCF7L2*-KO HCT116 tumors showed a similar increase in EPHA2 phosphorylation (Figure 2F). Notably, although total EPHA2 protein abundance showed only a minimal increase after short-term pharmacological Wnt inhibition (Figure 2E), long-term genetic Wnt inhibition via *TCF7L2* loss resulted in a pronounced elevation in total EPHA2 abundance (Figure 2F). RNA-Seq of HCT116 cells confirmed that *EPHA2* is the most highly expressed EPHA receptor in this model and that *EPHA2* transcript levels are upregulated in response to *TCF7L2* KO, corroborating the protein-level changes (Figure 2F and Supplemental Figure 2E). A similar induction of EPHA2 transcripts was also observed in *TCF7L2*-KO HT29 cells (Supplemental Figure 2F).

In the HPAF-II tumors, we examined additional EPHA2 phosphorylation sites as well. Wnt inhibition induced Y772 (activation loop) phosphorylation (34) with as little as 8 hours of treatment (Figure 2E). Interestingly, phosphorylation at the ligand-independent serine residue S897, often linked to protumorigenic signaling and enhanced cell motility, was also increased after ETC-159 treatment, suggesting that both ligand-dependent and ligand-independent EPHA2 pathways are simultaneously activated in response to Wnt inhibition in Wnt-addicted tumors (Figure 2E).

Our prior work indicated that Wnt inhibition drives several RTKs to the plasma membrane of cultured cells. IHC staining confirmed that pharmacological inhibition of Wnt signaling in HPAF-II xenograft tumors similarly drives EPHA2 to the plasma membrane (Figure 2G). Genetic inhibition of Wnt signaling by *TCF7L2* KO in HCT116 xenografts also drives EPHA2 to the plasma membrane (Figure 2H). In the appropriate tumor microenvironment, this may facilitate ligand engagement and receptor activation. Taken together, these findings identify EPHA2 as an abundant RTK activated in response to Wnt pathway inhibition.

### Loss of EPHA2 leads to nuclear accumulation of activated ERK.

There is substantial but conflicting literature on the interaction of EPHA2 and MAPK signaling (22, 24–27). We asked if EPHA2 activation might be responsible for Wnt inhibition–induced ERK phosphorylation in Wnt-addicted tumors. To test this, we knocked out *EPHA2* in HPAF-II cells using a 2-guide CRISPR strategy that resulted in the complete deletion of EPHA2’s kinase domain (deletion of exons 10–15) (Supplemental Figure 3A). Two independent *EPHA2*-KO clones (clones 18 and 21) (Supplemental Figure 3A) were then used to generate orthotopic xenograft tumors (Figure 3, A and D). KO of *EPHA2* led to a marked reduction in the intensity of the Wnt-regulated phosphotyrosine band at 140 kDa, consistent with EPHA2 being a major RTK phosphorylated after Wnt inhibition (Figure 3B). The residual signal likely reflects phosphorylation of other EPH family members.

We then tested whether *EPHA2* KO decreased MAPK activation. Contrary to our expectations, loss of EPHA2 in either clone markedly increased ERK phosphorylation in the orthotopic xenograft tumors (Figure 3, C and D, and Supplemental Figure 3B) to the same level achieved by Wnt inhibition alone. IHC analysis of orthotopic xenograft tissue sections confirmed that *EPHA2* KO caused both increased ERK phosphorylation and nuclear accumulation, similar to what was found with ETC-159 treatment (Figure 3E). Notably, *EPHA2* KO alone was sufficient to drive substantial nuclear localization of p-ERK, even in the absence of pharmacological Wnt inhibition (Figure 3E).

### Unexpected uncoupling of ERK activation and transcriptional response.

As shown in Figure 1, the phosphorylation of ERK induced by Wnt inhibition leads to robust upregulation of bona fide MAPK target genes (11) (Figure 1, A and D, and Supplemental Figure 1). We therefore asked if the ERK activation observed in *EPHA2*-KO tumors resulted in similar transcriptional changes. To this end, we performed RNA-Seq of the *EPHA2*-WT and -KO tumors. Using cutoffs of 1.5-fold change and adjusted *P* of 0.05 or less, *EPHA2* KO changed the expression of 357 genes, but despite robust nuclear accumulation of p-ERK, these tumors failed to upregulate the panel of previously determined bona fide MAPK target genes (Figure 3, F and G, Supplemental Table 3). Specifically, only 20 of the previously determined 179 MAPK target genes were upregulated by *EPHA2* KO. Using an independent MAPK target gene dataset generated from multiple cell lines in vitro (35), we determined only 24 of the EPHA2-regulated genes were MAPK targets. Thus, MAPK activated by EPHA2 loss alone does not recapitulate a robust MAPK transcriptional response. However, Wnt inhibition elicited a strong MAPK transcriptional response in both WT and *EPHA2*-KO tumors (Figure 3, F and G). Gene set enrichment analysis also showed that although the MAPK target genes were enriched upon Wnt inhibition in parental HPAF-II tumors, no such enrichment was observed in the untreated *EPHA2*-KO tumors (Supplemental Figure 3C). These findings were corroborated by real-time quantitative PCR (RT-qPCR) analysis of selected MAPK target genes in tumors derived from an independent *EPHA2*-KO clone (clone 21) that also exhibited elevated ERK phosphorylation (Figure 3, D and H). Taken together, these results indicate that *EPHA2* KO–induced p-ERK translocates to the nucleus but does not activate downstream transcription of our predefined gene set.

We previously showed that Wnt inhibition–induced MAPK activation is associated with the upregulation of senescence-associated genes and increased senescence-associated β-gal staining (11). These findings align with prior studies linking unchecked oncogenic MAPK signaling to senescence (29). Since *EPHA2*-KO tumors display high levels of nuclear p-ERK without concurrent MAPK transcriptional activation, we asked whether they would still exhibit a senescence-associated transcriptional profile. To address this question, we assessed the expression of 79 senescence-associated genes from the SenMayo gene set using RNA-Seq data from WT and *EPHA2*-KO tumors treated with or without ETC-159 (36). As expected, Wnt inhibition induced robust upregulation of a subset of senescence-associated genes in WT xenografts. Importantly, despite elevated ERK phosphorylation, the expression profile of *EPHA2*-KO tumors closely resembled that of vehicle-treated WT tumors and showed no upregulation or enrichment of ETC-159–responsive senescence genes (Figure 4, A and B, and Supplemental Figure 4A). However, Wnt inhibition triggered comparable changes in senescence gene expression in both WT and KO tumors, confirming that *EPHA2*-KO tumors retain transcriptional responsiveness to attenuated Wnt signaling (Figure 4, A and B). Consistent with these findings, baseline levels of senescence-associated β-gal staining were similar between *EPHA2*-KO and WT tumors, despite high levels of p-ERK in the *EPHA2*-KO tumors (Figure 4C). PORCN inhibition robustly increased senescence-associated β-gal staining in both groups, demonstrating that *EPHA2*-KO tumors remain fully competent to undergo senescence upon Wnt withdrawal.

Thus, robust phosphorylation of ERK activation sites after *EPHA2* KO induced neither MAPK target genes (Figure 3, F–H) nor the senescence-associated genes upregulated by Wnt inhibition (Figure 4, A and B, and Supplemental Figure 4A). However, the *EPHA2*-KO tumors remained responsive to Wnt inhibition, indicating that additional Wnt-dependent mechanisms gate the transcriptional output of nuclear p-ERK in this context.

### Wnt-regulated transcriptional repressors may constrain MAPK target gene expression.

The discrepancy in transcriptional responses between *EPHA2* KO–induced and ETC-159–induced p-ERK led us to hypothesize the existence of a Wnt-regulated transcriptional repressor that may restrict MAPK target gene activation even if upstream RTKs are active. This is consistent with our previous model that Wnt signaling dampens ERK-regulated transcription (11). To identify candidate repressors, we looked for transcription factors that are repressed by Wnt inhibition but not by *EPHA2* KO and are predicted to bind to the promoters of our 179 bona fide MAPK target genes as determined using ChIP-X Enrichment Analysis 3 (ChEA3).

Among the top candidates was GATA3, a zinc-finger transcription factor transcriptionally and posttranscriptionally regulated by the Wnt pathway (33, 37) (Figure 4, D and E). Western blot analysis confirmed a marked reduction in GATA3 protein levels after Wnt inhibition in both HPAF-II WT and *EPHA2*-KO tumors (Figure 4E). To assess whether GATA3 functionally represses MAPK target genes, we knocked down *GATA3* in *EPHA2*-KO cells. *GATA3* knockdown modestly increased the expression of selected MAPK targets, *WNT7A*, *DUSP5*, *EPHA4*, and *CST1* (Figure 4, F and G). Similarly, in the *GATA3* partial KO cells that we were able to obtain, we observed a moderate increase in selected MAPK target genes (Figure 4, H and I). This modest effect in vitro is likely due to low basal ERK activity in HPAF-II cells in vitro, particularly in the absence of extracellular EGFR and Eph-family ligands that are more abundant in vivo. Although preliminary, these findings support a model in which GATA3 and related transcriptional repressors limit MAPK target gene expression in the Wnt-high condition, with their downregulation upon Wnt inhibition facilitating a transcriptional response to ERK activation.

### EPHA2 KO activates EGFR to drive ERK phosphorylation.

Our xenograft studies utilizing 2 independent *EPHA2*-KO clones confirmed that loss of EPHA2 robustly activates ERK phosphorylation in Wnt-addicted pancreatic cancers (Figure 3). To understand how EPHA2 loss triggered ERK phosphorylation, we performed IP-MS on parental and *EPHA2*-KO xenograft tumors with or without PORCN inhibition. This analysis revealed that KO of *EPHA2* resulted in increased phosphorylation of EGFR at multiple tyrosine residues including Y1138, Y1172, and Y1197, several-fold higher than induced by Wnt inhibition alone (Figure 5, A and B, and Supplemental Figure 5A). This more robust activation of EGFR also correlated with increased phosphorylation of downstream Src-family kinases (Supplemental Figure 5B). Notably, the same EGFR sites were identified in our prior IP-MS analysis as being phosphorylated in response to Wnt inhibition in parental HPAF-II tumors (Figure 2C). Wnt inhibition in the *EPHA2*-KO tumors did not increase EGFR phosphorylation further (Figure 5B). These findings indicate that EPHA2 normally suppresses EGFR activation in tumors, consistent with previous reports showing repressive interaction between EPHA2 and components of the EGFR/MAPK pathway (25, 26).

Given that *EPHA2* KO activated both EGFR and Src-family kinases, we next asked whether the increased EGFR activity was the primary driver of activated ERK in the *EPHA2*-KO tumors. To test this, mice bearing *EPHA2*-KO tumors were treated with the EGFR inhibitor erlotinib, either alone or in combination with ETC-159. As expected, erlotinib treatment led to a marked reduction in EGFR phosphorylation at Y1068, a well-characterized activating residue (Figure 5C). Strikingly, ERK phosphorylation was also markedly reduced in *EPHA2*-KO tumors after erlotinib treatment, and concurrent Wnt inhibition did not restore ERK phosphorylation (Figure 5C). These findings indicate that the ERK activation observed in response to EPHA2 loss is largely driven by activated EGFR. In the *EPHA2*-KO tumors, erlotinib also decreased both basal and ETC-159–activated expression of a subset of MAPK target genes, consistent with inhibition of both EGFR and its downstream signaling kinases (Figure 5D).

Residual ERK phosphorylation was observed in tumors cotreated with ETC-159 and erlotinib (Figure 5C), which may be the consequence of several factors, including the presence of mutant RAS and membrane recycling of additional RTKs such as ERBB2, which was also detected in our IP-MS analysis (Figure 5A).

### Wnt inhibition works in large part via EGFR to activate ERK and MAPK target gene expression.

Our initial hypothesis was that Wnt inhibition turned on EPHA2 kinase activity, which in turn was responsible for increased ERK phosphorylation. However, *EPHA2* KO experiments disproved this, as loss of EPHA2 alone led to a robust and sustained increase in ERK phosphorylation, even in the absence of Wnt inhibition (Figure 3). This suggests that EPHA2 may function as a negative regulator of MAPK signaling, consistent with prior findings in breast and lung cancer models (25, 26). Given these results, we revisited the question of which RTK mediates the ERK activation in response to Wnt inhibition.

Our previous work demonstrated that Wnt inhibition increases EGFR surface abundance and phosphorylation in HPAF-II tumors (12), supporting a model in which EGFR is activated downstream of Wnt suppression. Our current IP-MS data revealed phosphorylation at multiple EGFR tyrosine residues (Y1138, Y1172, Y1197) after either Wnt inhibition or EPHA2 loss (Figure 2C and Figure 5, A and B), indicating that EGFR may serve as a common upstream regulator of ERK activation in both *EPHA2* KO and Wnt inhibition conditions.

To functionally assess EGFR’s role in Wnt-regulated MAPK activation, we treated orthotopic xenografts with vehicle, ETC-159, erlotinib, or the combination of ETC-159 and erlotinib. As expected, erlotinib treatment prevented EGFR phosphorylation (Figure 6A). The ERK activation caused by Wnt inhibition was partially attenuated by erlotinib cotreatment (Figure 6A). At the transcriptional level, the MAPK target genes induced by Wnt inhibition (e.g., *ARL14*, *WNT7A*) were also reduced by erlotinib (Figure 6, B and C). These findings indicate that EGFR contributes substantially, but not exclusively, to ERK activation and MAPK gene induction after Wnt inhibition in HPAF-II xenografts.

Consistent with a broader RTK response after Wnt inhibition, phosphotyrosine profiling also confirmed phosphorylation of ERBB2 at Y1248 after ETC-159 treatment (Figure 2C). Although we have not directly tested the functional role of ERBB2 in this setting, these observations support the model that Wnt inhibition allows a number of RTKs including EGFR, ERBB2, and EPHA2 to recycle to the plasma membrane, where a subset then further activate MAPK signaling even in the setting of mutant RAS.

### Wnt inhibition partially restores sensitivity to erlotinib.

HPAF-II tumors are driven by a combination of Wnt and KRAS(G12D) pathway activation. Consequently, treatment of HPAF-II xenograft–bearing mice with the EGFR inhibitor erlotinib (upstream of RAS) alone did not decrease tumor growth (Figure 6D) but, as previously reported, a low dose of ETC-159 modestly suppressed tumor growth (9). Notably, in the presence of low-dose ETC-159, erlotinib treatment resulted in a significant growth inhibition at 15 days of treatment (Figure 6D). This suggests that the activation of EGFR by Wnt inhibition promotes network rewiring and hence sensitivity to erlotinib (38). Attenuation of EGFR/RAS/MAPK signaling may also be more effective when layered onto a transcriptional landscape already downregulated by Wnt inhibition, which inhibits expression of genes involved in cell cycle control, DNA repair, and ribosome biogenesis (10, 16). Because Wnt inhibition activates more RTKs than EGFR alone, future studies can address whether additional RTK inhibitors may give more robust effects.

### Knocking out EPHA2 mildly accelerates tumor growth while enhancing sensitivity to both Wnt inhibition and erlotinib.

Given the unexpected absence of MAPK target gene induction despite robust nuclear p-ERK accumulation in *EPHA2*-KO tumors (Figure 3), we investigated whether this decoupling of ERK phosphorylation from transcriptional output had additional functional consequences in vivo. Tumor growth analysis revealed that *EPHA2*-KO tumors grew marginally faster than the WT tumors, perhaps by increased activation of both EGFR and additional kinases downstream of EGFR (Figure 7, A and B, and Supplemental Figure 5B). However, the growth of the *EPHA2*-KO tumors remained sensitive to Wnt pathway inhibition (30 mg/kg ETC-159) (Figure 7, A and B). Ki-67 and H&E staining confirmed that *EPHA2*-KO tumors were morphologically similar to the parental HPAF-II tumors and were highly proliferative (Figure 7C and Supplemental Figure 6A). Treatment with ETC-159 markedly reduced the number of Ki-67–positive cells, mirroring results observed in the parental HPAF-II tumors and underscoring the fact that EPHA2 loss does not impair responsiveness to Wnt inhibition.

We next examined the importance of the EPHA2/EGFR axis in regulating tumor growth. The *EPHA2*-KO tumors with activated EGFR became sensitive to EGFR inhibition with erlotinib (Figure 7D), again, likely due to activation of other kinases downstream of EGFR (Supplemental Figure 5B). What was especially striking was that the *EPHA2*-KO tumors now became much more sensitive to low-dose Wnt inhibition (10 mg/kg ETC-159) (Figure 7D compared with Figure 7A). This is consistent with the model that robust activation of MAPK signaling leads to differentiation and senescence, although other models are possible. Further study is needed to define this more precisely. Collectively, our results indicate that loss of EPHA2 and inhibition of Wnt signaling enhance the reliance of KRAS mutant Wnt-driven pancreatic cancers on EGFR signaling (Figure 7D). Additionally, the increased sensitivity of EPHA2-deficient tumors to both Wnt and EGFR inhibition suggests that EPHA2 loss may enhance RTK/MAPK and Wnt pathway dependencies due to signaling rewiring.

## Discussion

Building on our previous studies showing the role of Wnt/MAPK crosstalk (11) in regulating cell fate and tumor growth, we sought to identify the RTKs that contribute to ERK activation after Wnt pathway suppression. Our IP-MS analysis identified at least 2 major RTKs, EPHA2 and EGFR, whose activation was suppressed by Wnt signaling. EGFR is well recognized as a robust activator of MAPK signaling (28), whereas EPHA2 exhibits complex and context-dependent effects on MAPK pathway output (25–27).

In Wnt-high pancreatic tumors, we found that EPHA2 acts as a negative regulator of ERK phosphorylation, even in the presence of mutant KRAS. Notably, ERK activation resulting from EPHA2 loss did not result in induction of bona fide MAPK target genes, indicating that ERK phosphorylation is functionally decoupled from its expected transcriptional output. Notably, MAPK target genes were still robustly upregulated in *EPHA2*-KO tumors upon Wnt inhibition, implicating additional Wnt-dependent transcriptional factors in regulating MAPK-dependent gene expression (Figure 7E).

In contrast to EPHA2, we found that EGFR is a key upstream activator of MAPK target genes downstream of Wnt inhibition. Pharmacological inhibition of EGFR with erlotinib not only suppressed MAPK target gene expression but also combined with the Wnt inhibitor to prevent the growth of Wnt-high pancreatic cancers. Moreover, *EPHA2*-KO tumors also had elevated phosphorylation of EGFR and downstream signaling kinases, leading to increased sensitivity to EGFR inhibition. Collectively, these findings support a model in which EGFR functions as a central node for ERK activation after either Wnt pathway inhibition or EPHA2 loss (Figure 7E).

Previous studies have shown that EPHA2 upregulation can drive resistance to EGFR and HER2 inhibitors (23, 24). Consistent with this research, our work demonstrates that EPHA2 loss enhances sensitivity to erlotinib monotherapy in Wnt-high pancreatic cancer models. In contrast, increased EPHA2 expression has also been linked to enhanced ERK signaling in breast cancer models (27). Our observations align more closely with studies showing a negative regulatory relationship between EPHA2 and the MAPK pathway (25, 26). These opposing outcomes may reflect the context-dependent nature of EPHA2 signaling, which may depend on the inherent differences in RTK repertoires, ligand availability, and signaling across tumor types.

Several studies have shown interactions between EPHA2 and EGFR at multiple levels (22, 24–27). One study proposed that EPHA2 loss stabilizes the interaction between RAS and RAF within the MAPK cascade (26). Consistent with this study, we observed increased total c-RAF protein in *EPHA2*-KO tumors relative to parental HPAF-II controls (Supplemental Figure 7). However, phosphorylated c-RAF levels remained largely unchanged, suggesting that RAS-RAF stabilization alone is insufficient to account for downstream changes. The finding that erlotinib does not completely inhibit the expression of MAPK targets is consistent with our MS data showing that additional RTKs beyond EGFR are activated by EPHA2. Further studies will be needed to determine how EPHA2 loss drives EGFR and ERK activation, a process that might involve RTK heterodimerization, receptor recycling, and/or regulation by protein tyrosine phosphatases.

Although the link between EPHA2 loss and EGFR-driven MAPK activation has been explored (25, 39), relatively little attention has been paid to downstream transcriptional consequences. Here, we made the surprising observation that ERK activated by EPHA2 loss translocates to the nucleus, but it does not drive expression of many Wnt inhibition–responsive MAPK or senescence-associated genes. Intriguingly, these genes are robustly induced only when EPHA2 loss is combined with Wnt pathway inhibition, prompting us to hypothesize the existence of a Wnt-regulated transcription factor that gates MAPK output.

We identified GATA3 as a potential transcriptional gatekeeper. GATA3 expression is strongly downregulated by Wnt inhibition but not by EPHA2 loss. Previous studies have shown that GATA3 contains an FBXW7 phosphodegron allowing regulation by GSK3, a kinase central to Wnt/β-catenin signaling (37). Consistent with a gatekeeper effect, GATA3 interacts with members of the nucleosome remodeling and deacetylase (NuRD) complex G9A and MTA3 to repress gene expression in breast cancer (40). This finding aligns well with the Wnt-dependent repression of MAPK target genes in our study. Supporting this model, GATA3 knockdown via siRNA modestly upregulated selected MAPK target genes in cultured cells. The modest effect can be explained by the low basal ERK activity in KRAS-mutant HPAF-II cells in vitro. This difference can be attributed to the extracellular milieu, where EGFR and Eph ligands are present only in the in vivo setting. It would be ideal to test this in xenografts, but we were unable to obtain complete *GATA3*-KO cell lines. Collectively, these findings provide experimental support for GATA3 as a Wnt-dependent transcriptional gatekeeper that restricts MAPK output (Figure 7E).

GATA3 may be one of several Wnt-regulated repressors. Additional transcription factors such as FOSL1, FOSL2, and NR2F2 also have Wnt-dependent expression patterns. Notably, FOSL1 has previously been shown to repress MAPK target gene expression in specific contexts (28), raising the possibility that multiple Wnt-regulated factors act in concert to regulate MAPK-dependent gene expression.

Studies from Hogan and coworkers have shown that EPHA2 has a key role in epithelial surveillance against oncogenic transformation (22, 41). In their model, RASV12 mutant cells upregulate EPHA2, facilitating their detection by neighboring normal epithelial cells expressing ephrin-A ligands (42) and thus triggering contractile and repulsive responses to eliminate these transformed cells. Similarly, in pancreatic ductal adenocarcinomas, KRAS G12D mutant cells are eliminated from the normal epithelium in an EPHA2-dependent manner (22). Loss of EPHA2 allows KRAS mutant cells to persist and form premalignant lesions, increasing the likelihood of malignant progression and epithelial barrier invasion. Our findings suggest the possibility that this tumor-suppressive role of EPHA2 may in part be due to its inhibitory effect on EGFR and MAPK signaling.

In summary, our study provides a mechanistic insight into how Wnt signaling intersects with RTK-driven MAPK activation, revealing a role for EPHA2 as a negative regulator of ERK transcriptional response. Importantly, we also show that EGFR is a key driver of MAPK signaling upon Wnt inhibition and EPHA2 loss, creating a vulnerability in Wnt-high pancreatic cancers. Our finding that Wnt inhibition synergizes with EGFR inhibitors and that EPHA2 loss enhances sensitivity to both EGFR and Wnt pathway inhibitors provides opportunities for exploring therapeutic strategies targeting these axes.

## Methods

### Sex as a biological variable.

Our study predominantly examined male mice because they exhibited less variability in phenotype. Comparable findings were observed on the occasions when female mice were included.

### Tissue culture.

HPAF-II cells were obtained from American Type Culture Collection (ATCC) and maintained in MEM supplemented with 10% FBS (HyClone), 1% penicillin-streptomycin (Nacalai Tesque), 1% GlutaMAX, 1% sodium pyruvate (100 mM), and 1% HEPES buffer solution (1 M) (Life Technologies). HCT116 *TCF7L2*-KO and HT29 *TCF7L2-*KO cell lines, along with their respective parental controls, were provided by Andreas Hecht, University of Freiburg, Freiburg, Germany (32). These cell lines were maintained under identical MEM culture conditions. All cell lines were cultured in a 37°C incubator with 5% CO_2_ and routinely tested for mycoplasma contamination, with all lines confirmed to be free of mycoplasma.

### Western blot analysis.

For immunoblot analysis, snap-frozen tumor fragments were homogenized in 4% SDS buffer using a Polytron homogenizer (IKA T10 basic Ultra-Turrax) set to a speed of 5. Equal amounts of protein (approximately 30 μg) were loaded onto 10% SDS-polyacrylamide gels and electrophoresed at 100 V for approximately 2 hours in Tris-glycine-SDS buffer (1st BASE) until the dye front had run out. Proteins were then transferred to PVDF membranes at 30 V for 16 hours using Tris-glycine transfer buffer (1st BASE) in a cold room at 4°C.

Membranes were probed with primary antibodies from Cell Signaling Technology using the recommended diluents (5% milk in PBST or 5% BSA in PBST) and concentrations (generally 1:1,000, though 1:2,000 was used for p-ERK). The primary antibodies used included p-p44/42 MAPK (Erk1/2) (Thr202/Tyr204) (catalog 9106), p44/42 MAPK (Erk1/2) (catalog 9102), EPHA2 (D4A2) XP (catalog 6997), p-EPHA2 (Tyr588) (D7X2L) (catalog 12677), p-EGF receptor (Tyr1068) (D7A5) XP (catalog 3777), p-tyrosine (P-Tyr-1000) MultiMab (catalog 8954), p-EPHA2 (Ser897) (D9A1) (catalog 6347), p-EPHA2 (Tyr772) (catalog 8244), GATA-3 (D13C9) (catalog 5852), p-c-Raf (Ser338) (56A6) (catalog 9427), c-Raf (D5X6R) (catalog 12552), β-actin (catalog 4967), and GAPDH (14C10) (catalog 2118).

After primary antibody incubation, blots were incubated with the appropriate HRP-conjugated secondary antibodies (mouse 1706516 or rabbit 1706515 from Bio-Rad), diluted at 1:10,000 in 5% milk in PBST, for 1–2 hours at room temperature. Membranes were developed using SuperSignal West Femto or SuperSignal West Dura chemiluminescent substrate (Thermo Fisher Scientific) and imaged using an Image Quant LAS 4000 camera system.

### Animal care.

NSG mice (NOD.Cg-*Prkdc^scid^ Il2rg^tm1Wjl^*/SzJ) were purchased from The Jackson Laboratory. The mice were housed in standard cages under controlled environmental conditions and provided ad libitum access to food and water.

### Animal studies.

Mouse xenograft models were established by injecting 1 × 10^6^ cells, resuspended in 50% Matrigel, into the pancreas of NSG mice as previously described (12).

For tumor growth experiments, 5 × 10^6^ HPAF-II or *EPHA2*-KO cells were suspended in 50% Matrigel and injected subcutaneously into both flanks of NSG mice. Once tumors were established, mice were treated with either ETC-159 or erlotinib as appropriate. ETC-159 and erlotinib were formulated in 50% PEG400 (vol/vol) in water and administered by oral gavage at a dosing volume of 10 μL/g of body weight. Tumor dimensions were measured using a digital vernier caliper, and volumes were calculated using the following equation: 0.5 × length × width^2^.

Mice were euthanized 8 hours after the final drug dose using carbon dioxide asphyxiation. After euthanasia, tumors were removed and processed for downstream analysis by snap-freezing in liquid nitrogen, fixation in 10% neutral-buffered formalin, or embedding in Tissue-Tek OCT compound.

### Cell lysate preparation for MS proteomics.

Approximately 16 mg of tumor tissue from each mouse was chopped on ice and mixed with disruption beads (Research Products International) in a 1:3 ratio, along with 400 μL of sodium deoxycholate lysis buffer (Merck). Samples were homogenized using a Bead Ruptor 12 (OMNI International) for 20 seconds at maximum speed, cooled on ice, and the process was repeated for 3 cycles. The resulting lysates were heated at 95°C for 5 minutes, followed by sonication at 10 amps for 1 minute, repeated 3 times, and then spun down to collect the supernatant. Next, 10 μL aliquot of each supernatant was transferred to a new 1.5 mL Eppendorf tube for protein quantitation using the bicinchoninic acid assay (Pierce BCA protein assay kit, Thermo Fisher Scientific) according to the manufacturer’s instructions.

### Protein digestion.

The digestion step followed the EasyPhos protocol (43). Briefly, normalized lysates were denatured and alkylated simultaneously using a buffer containing 10 mM Tris(2-carboxyethyl)phosphine hydrochloride (Merck) and 40 mM 2-chloroacetamide (Merck). Lys-C (Wako Chemicals) and trypsin (Promega) were added at an enzyme-to-protein ratio of 1:100 (w/w), and digestion was carried out overnight at 37°C with shaking. Upon completion, the peptide solution was divided into 2 parts for phosphoproteome (PP) and tyrosine phosphorylation (YP) profiling. A defined volume of peptides (equivalent to 500 μg starting protein) was mixed with isopropanol for PP profiling. The remaining lysates were acidified with trifluoroacetic acid (TFA, Merck) to 2% (v/v) to remove sodium deoxycholate for YP profiling. After vortexing for 1 minute and centrifugation at 15,000*g* for 10 minutes, the supernatant was retained. This acid-mediated cleanup step was then repeated.

### Phosphopeptide enrichment and desalting.

For PP analysis, the peptide mixtures with isopropanol described above were combined with EasyPhos enrichment buffer and centrifuged to collect the supernatants (43). Samples were incubated with titanium dioxide beads (GL Sciences) at a 12:1 (w/w) bead-to-protein ratio for 5 minutes at 40°C with shaking at 2,000 rpm, followed by 4 washes. Phosphopeptides were eluted from the beads and dried in a SpeedVac. The dried peptides were resuspended onto Empore SDB-RPS StageTips (Sigma-Aldrich), eluted with StageTip elution buffer, dried completely using a SpeedVac, and finally resuspended in 8 μL of MS loading buffer for analysis.

### Phosphorylated Tyr peptide preparation.

A defined volume of each peptide mixture (equivalent to 20 mg of protein starting material) was loaded onto ACN-activated Sep-Pak columns, washed with 12 mL of 0.1% TFA, and eluted with 50% ACN and 0.2% TFA (v/v). The eluates were frozen and lyophilized for 1–2 days. Meanwhile, Rec-Protein G Invitrogen beads and antibodies were prepared at a 1:4 (v/w) ratio. Briefly, the beads were incubated with 50 μL phospho-Tyr-1000 antibody (Cell Signaling Technology) and 50 μL phospho-Tyr-20 antibody (BD Biosciences) in 1.7 mL of IP binding buffer (50 mM MOPS, 10 mM Na_2_HPO_4_, 50 mM NaCl, pH 7.5) overnight at 4°C. The next day, the antibody-coupled beads were washed 3 times with 1.8 mL of immunoaffinity purification (IAP) washing buffer (50 mM Tris-HCl, 150 mM NaCl, 1% *n*-octyl-β-D-glucopyranoside, pH 7.4) and transferred into new 2 mL Eppendorf tubes. The lyophilized peptides, dissolved in 1.8 mL of IAP washing buffer (adjusted to neutral pH), were added to the bead slurry and incubated overnight at 4°C. The beads were washed 3 times with 1 mL of IAP washing buffer, followed by 3 washes with 1 mL of Milli-Q water. After the final wash, phosphorylated tyrosine peptides were eluted using 55 μL of 0.15% trifluoroacetic acid with shaking at 1,400 rpm for 15 minutes at room temperature. The supernatant was collected, and the elution step was repeated to obtain a final volume of 110 μL. The phosphorylated tyrosine peptides were then desalted following the SDB-RPS StageTips protocol used for PP and resuspended in MS loading buffer for MS analysis.

### MS-based (phospho)proteome analysis.

MS samples were analyzed on an UltiMate 3000 RSLC nano-LC system (Thermo Fisher Scientific) coupled to a Q Exactive HF Mass Spectrometer (Thermo Fisher Scientific). Peptides were loaded via a PepMap100 trap column (100 μm × 2 cm, nanoViper; Thermo Fisher Scientific), and then eluted and separated on a PepMap100 RSLC analytical column (75 μm × 50 cm, nanoViper, C18; Thermo Fisher Scientific). For each injection, peptides were eluted using a 120-minute gradient from 2.5% to 42.5% buffer B (80% ACN, 0.1% FA), followed by a 6-minute wash with 99% buffer B, at a flow rate of 250 nL/min. The eluent was nebulized and ionized using a nano-electrospray source (Thermo Fisher Scientific) with a distal-coated fused silica emitter (Trajan).

The mass spectrometer was operated in data-dependent acquisition (DDA) mode to automatically switch between full MS and tandem MS/MS scans. Survey full-scan MS spectra (*m*/*z* 375–1575) were acquired in the Orbitrap at 60,000 resolution (at *m*/*z* 200) after accumulation to a target value of 1 × 10^6^ ions, with a maximum injection time of 54 ms. Up to 30 of the most intensely charged ions were sequentially isolated and fragmented in the collision cell using higher-energy collisional dissociation (HCD) with a fixed injection time of 120 ms, 30,000 resolution, and an automatic gain control target of 1 × 10^5^. Dynamic exclusion was set to 30 seconds.

### Data searching.

All raw profiling files from DDA acquisition were analyzed using MaxQuant (version 2.0.0.2) to identify phosphorylated peptides (44). Database searching was performed with the following parameters: carbamidomethylation as a fixed modification; oxidation (Met), N-terminal acetylation, and phosphorylation (Ser, Thr, Tyr) as variable modifications; up to 2 missed cleavages allowed; and a 1% FDR applied to both protein and peptide identifications. Human protein sequence databases were downloaded in 2022 from https://www.uniprot.org/

### Data processing and differential analysis.

The R package *PHOSR* was used to generate a matrix of phosphorylation sites, including normalization and missing value imputation (45). Differential analysis across sample subsets was performed using the R package limma. To control the FDR for multiple comparisons, *P* values were adjusted using the Benjamini-Hochberg method. An FDR-adjusted *P* value less than 0.05 was considered statistically significant. For figures in the main text, we plotted peptides with less than 2 imputed values. The full dataset is included in Supplemental Table 1.

### RNA isolation and reverse transcription quantitative PCR.

RNA was isolated from tumors using the RNeasy kit (QIAGEN) according to the manufacturer’s instructions. Briefly, 600 μL of RLT buffer containing β-ME (10 μL β-ME per 1 mL RLT) was added to a small piece (<30 mg) of flash-frozen tumor tissue. The tissue was homogenized using a Polytron homogenizer (IKA T10 basic Ultra-Turrax) set to speed 5, then transferred to a QIAshredder column and centrifuged at maximum speed for 2 minutes at 20°C–25°C. An equal volume (600 μL) of 70% ethanol was added to the flowthrough, and the mixture was transferred to an RNeasy Mini Spin Column in 2 portions. Each portion was centrifuged for 1 minute at more than 8,000*g* at room temperature. The flow-through was discarded, and 700 μL of RW1 buffer was added to the column, followed by centrifugation under the same conditions. This was followed by 2 washes with 700 μL of RPE buffer. The column was then placed in a new Eppendorf tube and centrifuged for 2 minutes to facilitate drying. RNA was eluted in 50–75 μL of RNase-free water and stored on ice. RNA purity and concentration were assessed using a NanoDrop spectrophotometer. For RNA obtained from cell culture, 350 μL of RLT buffer was used instead of 600 μL, and the QIAshredder step was omitted. All other steps remained unchanged.

cDNA was prepared in a 20 μL reaction using 1,000 ng of RNA (quantified by NanoDrop), 5 μL of MiRXES RT buffer, and 1 μL of MiRXES RT enzyme, as part of the BlitzAmp cDNA Synthesis kit (MiRXES, 1203101), or using iScript RT Supermix (Bio-Rad). Thermal cycling conditions were as follows: 42°C for 30 minutes, followed by 95°C for 5 minutes for the MiRXES reaction; and 25°C for 5 minutes, 46°C for 30 minutes, and 95°C for 1 minute for the iScript reaction.

RT-qPCR was performed using BlitzAmp qPCR Master Mix (MiRXES, 1204202). Gene expression was normalized to EPN1. Primer sequences are provided in Supplemental Table 4.

### IHC.

Sections of FFPE tumors were deparaffinized in xylene and rehydrated through a graded ethanol series. Antigen retrieval was performed in sodium citrate buffer (pH 6.0) by microwaving at boiling temperature for 10 minutes. Endogenous peroxidase activity was blocked by incubation with 3% hydrogen peroxide in TBS. Sections were incubated overnight at 4°C with p-p44/42 MAPK (Erk1/2) antibody (1:200 dilution, 4376S, Cell Signaling Technology) diluted in 3% BSA-TBST. After washing, slides were incubated with HRP-conjugated anti-rabbit secondary antibody (Bio-Rad, 1706515, diluted 1:200) for 1 hour at room temperature. Staining was visualized using DAB chromogen substrate, and nuclei were counterstained with Mayer’s hematoxylin. Brightfield images were acquired using a Nikon Eclipse Ni-E microscope.

For total EPHA2 staining, antigen retrieval was performed in 0.001 M EDTA (pH 8.0) for 18 minutes, followed by overnight incubation at 4°C with EPHA2 (D4A2) rabbit mAb (6997, Cell Signaling Technology) and detection using the Dako REAL EnVision Detection System, Peroxidase/DAB^+^, Rabbit/Mouse (K5007).

For Ki-67 staining, antigen retrieval was performed in sodium citrate buffer (pH 6.0) for 30 minutes, followed by overnight incubation at 4°C with Ki-67 (MM1) mouse mAb (1:100, Leica Biosystems) and HRP-conjugated anti-mouse secondary antibody (Bio-Rad, 1706516; 1:200) for 1 hour at room temperature.

### CRISPR-mediated gene KO.

*EPHA2* KO in HPAF-II cells was performed using a ribonucleoprotein–based (RNP-based) CRISPR/Cas9 electroporation strategy. Two sgRNAs targeting flanking regions of the EPHA2 kinase domain were individually complexed with Cas9 protein (*Streptococcus* *pyogenes*, IDT) in buffer R (Neon Transfection System, Invitrogen), forming 2 RNPs. These were combined in a 1:1 ratio prior to electroporation. HPAF-II cells at approximately 60% confluence were harvested using TrypLE Express (Gibco), resuspended in buffer R, and electroporated using the Neon system at 1150 V, 30 ms pulse width, and 2 pulses. After electroporation, cells were plated in complete EMEM and maintained under standard culture conditions.

KO efficiency was initially assessed by PCR amplification of genomic DNA using primers flanking the targeted approximately 3.4 kb region. A 351 bp product indicated successful deletion of the EPHA2 kinase domain, while the intact allele produced a 3.7 kb band. Clonal lines were further validated by Western blotting using an anti-EPHA2 antibody (Cell Signaling Technology, 6997), confirming loss of EPHA2 protein expression.

A GATA3 partial KO line in HPAF-II cells was generated using the same 2-guide RNP-based CRISPR/Cas9 electroporation strategy, with sgRNAs targeting exon 2 and intron 4 of *GATA3*. sgRNA sequences for EPHA2 and GATA3 are provided in Supplemental Table 4.

### RNA-Seq analysis.

Total RNA from tumors across 4 experimental conditions: HPAF-II WT vehicle (*n* = 4), HPAF-II WT plus ETC-159 56 hours (*n* = 5), *EPHA2* KO vehicle (*n* = 5), and *EPHA2* KO plus ETC-159 56 hours (*n* = 5) was submitted for poly(A)-selected RNA-Seq with a target depth of 60 million paired-end reads per sample. Raw sequencing quality was assessed using FastQC (Babraham Bioinformatics). Adapter trimming and low-quality read removal were performed using Trimmomatic v0.39 (46). To remove mouse-derived reads, samples were filtered against the GRCm39 genome using Xenome, as previously reported (16). The remaining human reads were aligned to the GRCh38.p7 reference genome (Ensembl release 85) using STAR v2.7.11b, as previously reported (16), and gene-level counts were obtained.

Because of read loss during graft-host filtering, most samples consisted of paired-end reads, although some contained a minority of single-end reads. For count-based analyses, paired-end and single-end counts were summed per gene. Gene-level transcripts per million (TPM) values were calculated from paired-end reads only and used to empirically filter low-expression genes. Genes with a median TPM less than 1 across all samples were excluded prior to differential expression analysis.

Differential gene expression was performed on raw counts using DESeq2 (https://bioconductor.org/packages/release/bioc/html/DESeq2.html), with independent filtering disabled. Genes with fewer than 100 total counts across all samples or without annotation were excluded. An FDR threshold of 0.05, adjusted via the Benjamini-Hochberg method, was used to define statistical significance. Log fold-change shrinkage was applied using the adaptive shrinkage estimator from the ashr package (47).

Read distribution and gene body coverage analyses were performed as part of post-alignment quality control. Over 90% of reads per sample aligned uniquely and mapped predominantly to exonic regions, with even coverage across housekeeping genes. The processed TPMs are in Supplemental Table 3.

### Data visualization in R.

Volcano plots and heatmaps were generated in R (v4.4.1) using a combination of CRAN and Bioconductor packages. Heatmaps were constructed using the pheatmap and heatmaply packages, with row-wise scaling and complete linkage hierarchical clustering. For phosphoproteomic datasets, gene names and phosphorylation site positions were concatenated and set as row identifiers. Gene expression heatmaps were created from log_2_-transformed TPM values, using a pseudocount of 0.01 to avoid zero values.

Volcano plots were generated using the EnhancedVolcano package, with gene symbols as labels and log_2_ fold-change and adjusted *P* values as axes. Custom thresholds for fold change and significance were applied depending on the dataset and are specified in the corresponding figure legends. Additional visualization and data manipulation were performed using the tidyverse and ggplotify packages.

### Senescence-associated β-gal staining.

Senescence-associated β-gal staining was performed on 8–10 μm cryosections obtained from OCT-embedded, snap-frozen orthotopic tumor tissues. Sections were fixed for 5 minutes at room temperature using the fixative solution provided in the Senescence Detection kit (Abcam, ab65351), followed by 3 brief washes in PBS containing 2 mM MgCl_2_. Staining was carried out using the kit’s X-gal solution according to the manufacturer’s instructions, with incubation at 37°C in a humidified chamber. The reaction was monitored microscopically and stopped upon the appearance of blue staining.

Sections were counterstained with Nuclear Fast red (Sigma-Aldrich, N3020) and mounted using glycerol-gelatin mounting medium. Brightfield images were acquired using a Nikon Eclipse Ni-E microscope equipped with a DS-Ri2 camera.

### siRNA-mediated knockdown of GATA3.

GATA3 was silenced in *EPHA2*-KO cells using a QIAGEN siRNA (si7) transfected with Lipofectamine RNAiMAX (Thermo Fisher Scientific) at a 1:1.5 ratio (siRNA/transfection reagent) in OptiMEM. Cells were seeded at 2.5 × 10^5^ cells per well in 6-well plates and transfected the following day with a final siRNA concentration of 100 nM in a total volume of 2 mL per well. Cells were harvested 48 hours after transfection for RNA isolation.

### Gene set enrichment analysis.

Gene set enrichment analysis was performed using the Broad Institute desktop application with ENSEMBL ID–based input files, including phenotype and expression matrices formatted to software specifications. Analyses used the KRAS upregulated hallmark gene set and the SenMayo senescence-associated gene set (36).

### Statistics.

For RT-qPCR data, statistical analysis was performed on ΔCq values using unpaired, 2-tailed Mann-Whitney *U* tests in GraphPad Prism. A *P* value less than 0.05 was considered statistically significant.

### Study approval.

All procedures involving mice were reviewed and approved by the SingHealth IACUC, Singapore.

### Data availability.

The MS proteomics data have been deposited to the ProteomeXchange Consortium via the PRIDE (48) partner repository with the dataset identifier PXD079682 (https://www.ebi.ac.uk/pride/archive/projects/PXD079682), and are also presented in Supplemental Table 1. The RNA-Seq data are available at NCBI’s BioProject with identifier PRJNA1478800 (https://ncbi.nlm.nih.gov/bioproject/?term=PRJNA1478800), and in Supplemental Table 3. Values for all data points in graphs are reported in the Supporting Data Values file and Supplemental Table 5.

## Author contributions

SRW, RJD, BM, and DMV designed the study. SW, BM, SP, and SS performed animal studies and biochemical assays. CH and RJD performed and analyzed the phosphoproteomic assays. BM and DMV supervised the study. SW, BM, and DMV wrote the manuscript with significant input from RJD. All authors read and approved the manuscript.

## Conflict of interest

DMV and BM have a financial interest in the Wnt inhibitor ETC-159.

## Funding support

- National Research Foundation Singapore STAR Award MOH-000155 administered by the Singapore Ministry of Health’s National Medical Research Council (to DMV).
- Singapore Ministry of Health’s National Medical Research Council Open Fund-Independent Research grant NMRC/OFIRG/0055/2017 (to BM).

## Supplementary Material

Supplemental data

Unedited blot and gel images

Supplemental table 1

Supplemental table 2

Supplemental table 3

Supplemental table 4

Supplemental table 5

Supporting data values

## Figures and Tables

**Figure 1:**
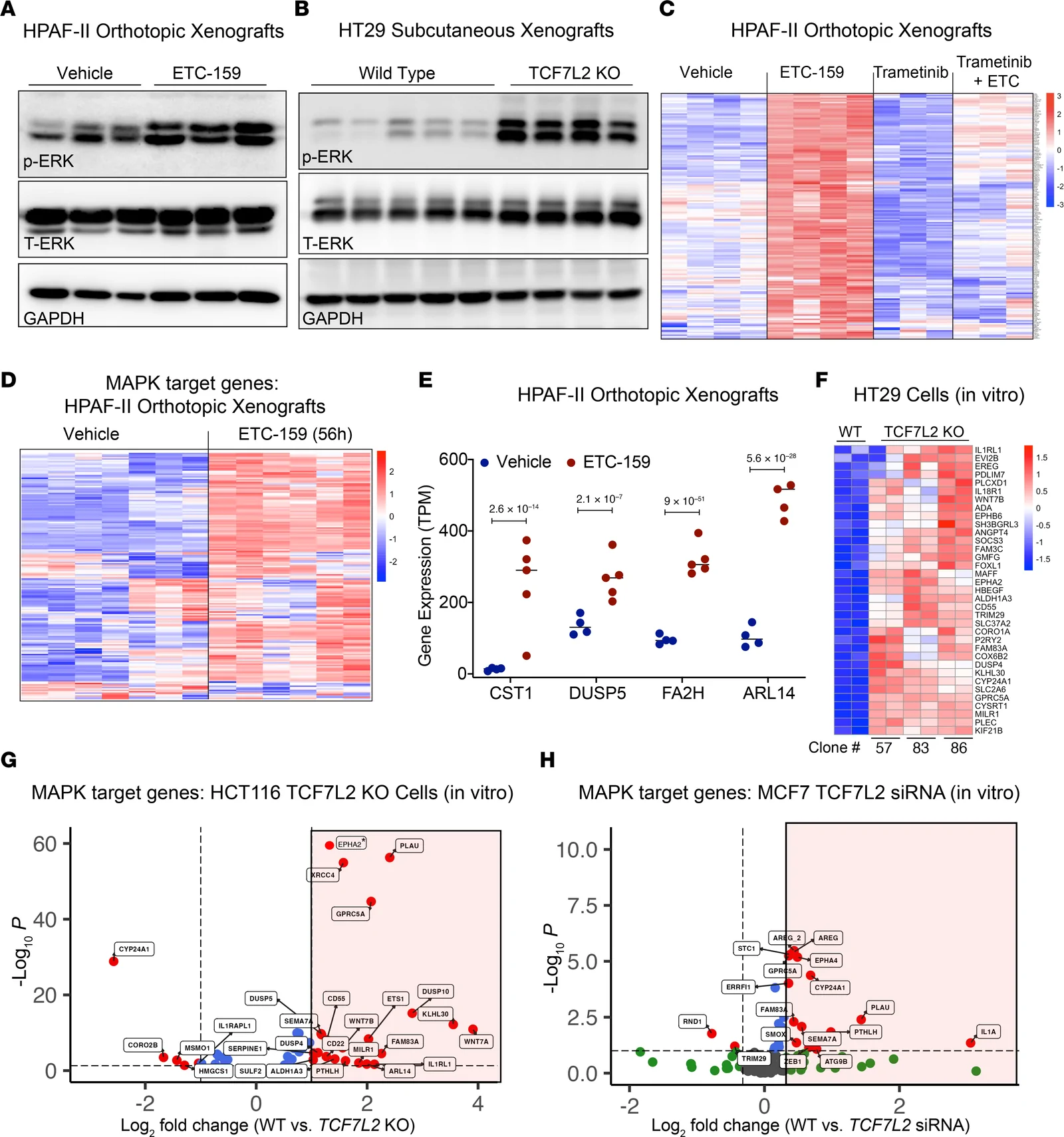
Pharmacological and genetic inhibition of Wnt signaling triggers ERK phosphorylation and upregulation of MAPK target genes. (**A**) Pharmacological inhibition of Wnt signaling induces ERK phosphorylation in HPAF-II orthotopic xenografts after 4 hours of ETC-159 treatment. Each lane represents tumor lysate from an individual tumor. (**B**) Genetic inhibition of Wnt signaling via *TCF7L2* KO increases ERK phosphorylation in HT29 subcutaneous xenografts (*n* = 4–6 mice/group). (**C**) MAPK target set was defined as genes whose induction by ETC-159 was attenuated by 50% or more upon cotreatment with the MEK inhibitor trametinib. RNA-Seq analysis of HPAF-II orthotopic xenografts identified 179 such genes. Heatmap shows their scaled differential expression (*z* scores). (**D** and **E**) Wnt inhibition with ETC-159 upregulates MAPK target gene expression in HPAF-II orthotopic tumors. (**D**) Heatmap shows *z* scores for 179 bona fide MAPK targets (*n* = 6–7 mice/group). (**E**) Representative MAPK target genes selected from **D**. Expression shown as mean ± SD; TPM, transcripts per million. Adjusted *P* values were calculated using the Benjamini-Hochberg method. (**F** and **G**) Genetic inhibition of Wnt signaling via *TCF7L2* KO increases MAPK target gene expression in HT29 and HCT116 colon cancer cells. (**F**) Heatmap shows *z* scores for 35 bona fide MAPK targets in parental HT29 and 3 *TCF7L2*-KO clones (nos. 57, 83, and 86). (**G**) Volcano plot shows differential expression of bona fide MAPK targets in *TCF7L2*-KO versus parental HCT116 cells. *(log_2_ fold change = 1.32; adjusted *P* = 1.97 × 10^–195^). Initial data from Wenzel et al. (32). (**H**) Knockdown of *TCF7L2* upregulates MAPK target genes in MCF7 breast cancer cells. Volcano plot shows differential expression of bona fide MAPK targets in *TCF7L2* siRNA–treated versus control siRNA–treated cells. A log_2_ fold-change threshold of 0.322 (equivalent to 1.25-fold change) was applied. Initial data from Frietze et al. (33).

**Figure 2:**
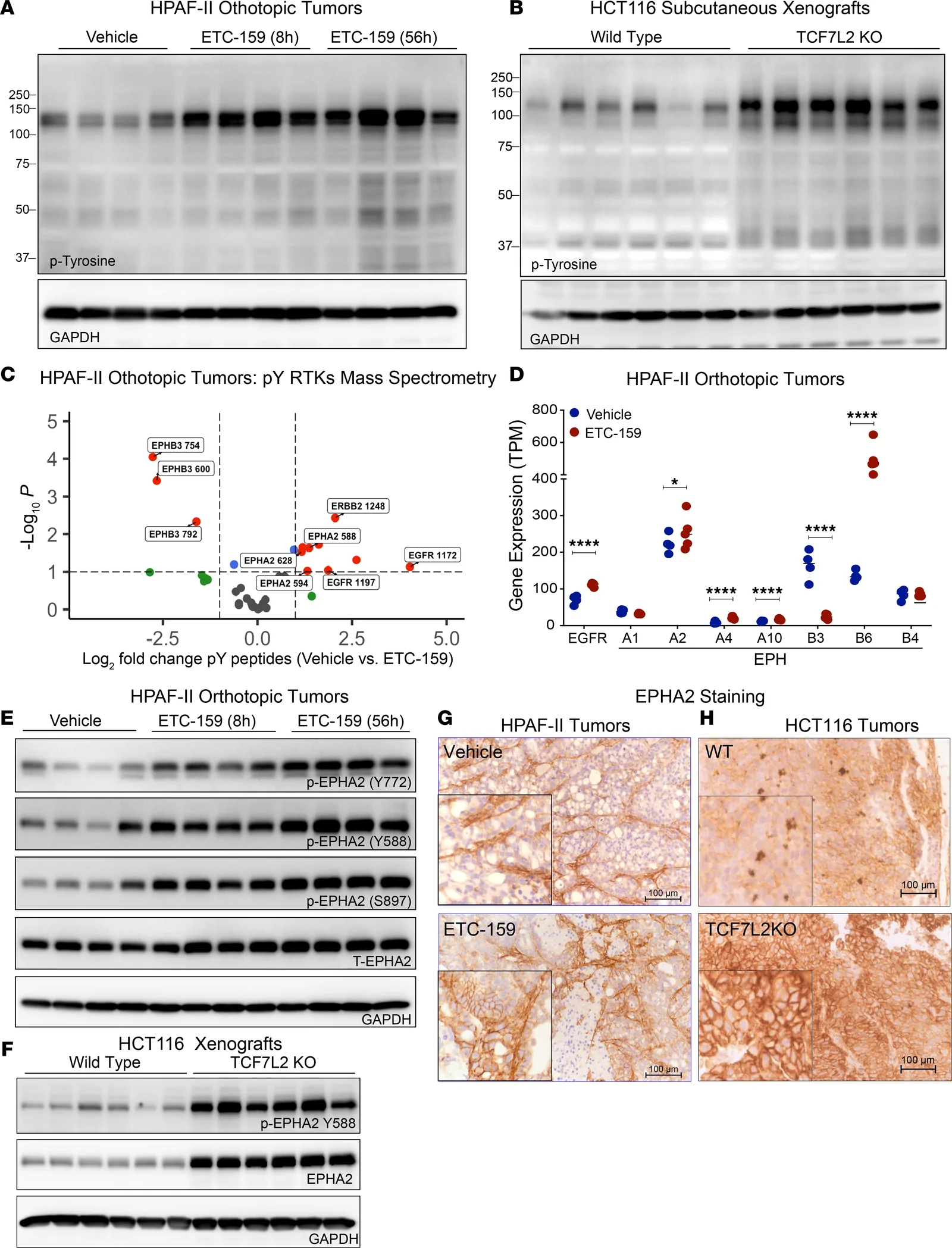
Wnt inhibition induces phosphorylation of specific RTKs including EPHA2, EGFR, and ErbB2. (**A**) Pharmacological Wnt inhibition increases tyrosine phosphorylation in HPAF-II orthotopic xenografts. Tumor lysates from mice treated with ETC-159 for 8 or 56 hours (*n* = 4 mice/group) were probed with anti-phosphotyrosine antibody PY1000. (**B**) Genetic inhibition of Wnt signaling via *TCF7L2* KO increases tyrosine phosphorylation in HCT116 subcutaneous xenografts (*n* = 6 mice/group). (**C**) Wnt inhibition promotes phosphorylation of specific RTKs, including EPHA2 and EGFR. Volcano plot depicts differential phosphotyrosine enrichment of RTKs in HPAF-II orthotopic xenografts after 56 hours of ETC-159 treatment. Data were obtained by phosphotyrosine IP-MS using PY20 and PY1000 antibodies. (**D**) RTK expression in HPAF-II orthotopic xenografts. *EPHA2* is the most highly expressed EphA receptor in HPAF-II tumors and is modestly upregulated after Wnt inhibition. EPHB6 is kinase dead (*n* = 4–5 mice/group). *EGFR* is also highly expressed and modestly induced. Benjamini-Hochberg–adjusted *P* values are shown (adjusted *P < 0.01 and ****P < 0.0001). (**E**) ETC-159 treatment induces phosphorylation of EPHA2 at multiple residues in HPAF-II xenografts. Immunoblot shows increased phosphorylation at Y588, Y772, and S897 after 8 or 56 hours of ETC-159 treatment (*n* = 4 mice/group). (**F**) Genetic inhibition of Wnt signaling via *TCF7L2* KO increases EPHA2 phosphorylation in HCT116 xenografts. Immunoblot shows increased p-EPHA2 (Y588) and total EPHA2 abundance in *TCF7L2*-KO versus control HCT116 tumors (*n* = 6 mice/group). (**G** and **H**) Wnt inhibition increases cell surface abundance of EPHA2. IHC of EPHA2 in (**G**) HPAF-II orthotopic xenografts treated with vehicle or ETC-159 (56 hours) and (**H**) HCT116 parental and *TCF7L2*-KO xenografts. Scale bars: 100 μm.

**Figure 3:**
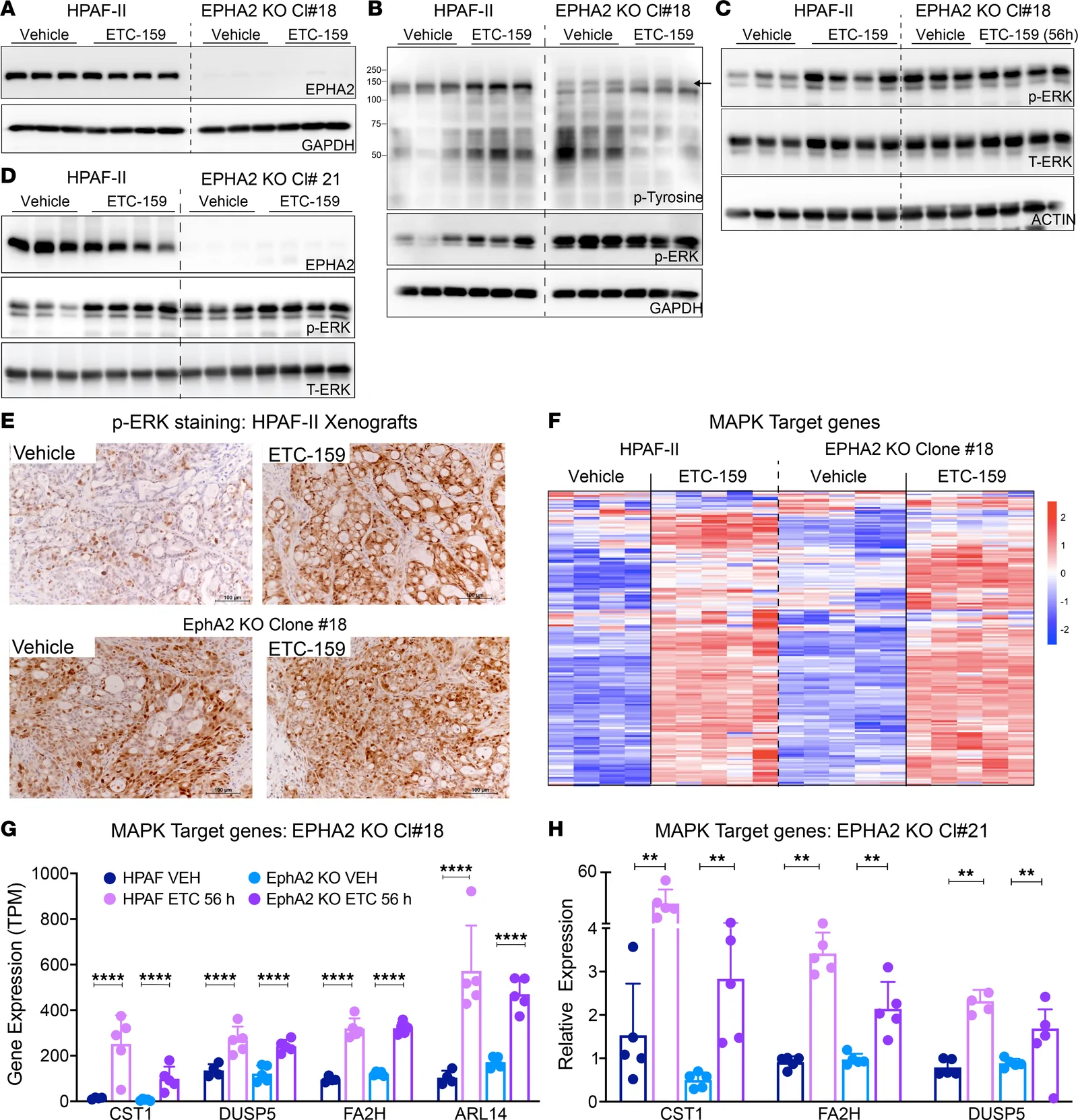
*EPHA2* KO induces ERK phosphorylation without concomitant upregulation of MAPK target genes. (**A**) *EPHA2* KO efficacy. Each lane represents an individual parental HPAF-II or *EPHA2*-KO orthotopic xenograft from mice treated with vehicle or ETC-159 for 8 hours. (*n* = 3–4 mice/group). (**B**) Top: *EPHA2* KO reduces phosphotyrosine signal at approximately 140 kDa (arrow), consistent with EPHA2 being a major contributor to tyrosine phosphorylation at this molecular weight. ETC-159 does not restore this band in *EPHA2*-KO tumors (*n* = 3 mice/group, 8-hour treatment). Bottom: *EPHA2* KO markedly increases basal ERK phosphorylation (T202/Y204) with no further enhancement by ETC-159 treatment. (**C**) *EPHA2*-KO tumors have elevated p-ERK levels comparable to the ETC-159–treated parental HPAF-II tumors, with no further increase after ETC-159 treatment, indicating that EPHA2 loss alone drives maximal ERK activation at baseline (*n* = 4–6 mice/group). (**D**) Increased ERK phosphorylation was confirmed using an independent *EPHA2*-KO clone 21 (3–4 mice/group). (**E**) *EPHA2* KO induced p-ERK translocation to the nucleus. Representative p-ERK IHC images of tissue sections from parental and *EPHA2*-KO orthotopic tumors treated with vehicle or ETC-159 for 56 hours. Scale bars: 100 μm. (**F**) Loss of EPHA2 alone (with concomitant phosphorylation of ERK) does not alter MAPK target gene expression, whereas Wnt inhibition induces these genes in *EPHA2*-KO xenografts. Heatmap of *z* scores for 179 bona fide MAPK target genes from Figure 1C (*n* = 4–5 mice/group; 56 hours of ETC-159 or vehicle treatment). (**G**) Representative MAPK target genes from **F**. Benjamini-Hochberg–adjusted *P* values are shown (adjusted ****P < 0.0001). (**H**) Lack of MAPK target gene expression induced by *EPHA2* KO alone was confirmed in independent clone 21. mRNA abundance determined by RT-qPCR (*n* = 5 mice/group, 56 hours’ treatment with ETC-159 or vehicle). *P* values determined by unpaired, 2-tailed Mann-Whitney *U* test on ΔCq values; **P < 0.01.

**Figure 4:**
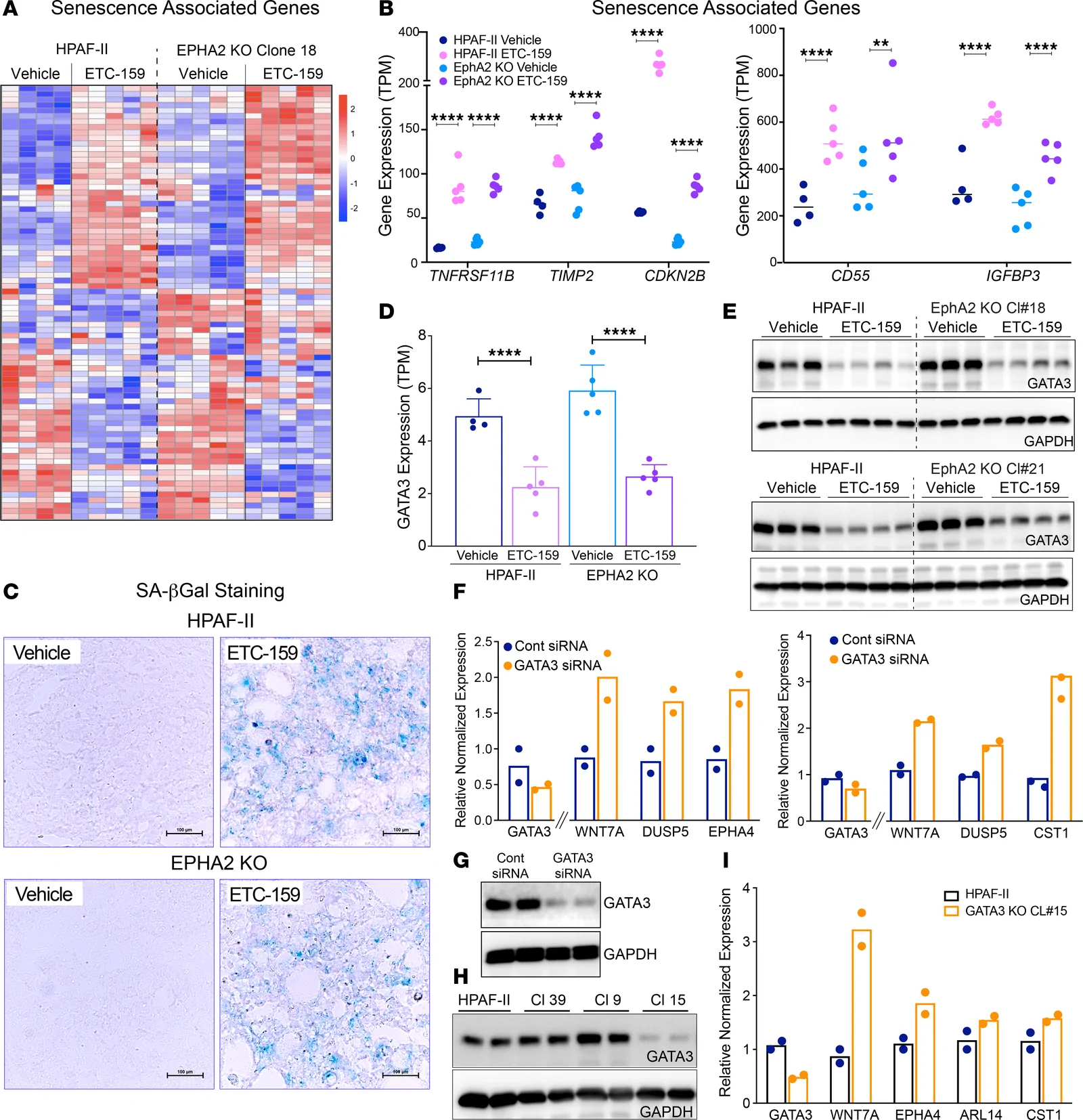
*EPHA2* KO–induced ERK phosphorylation does not elicit senescence, and GATA3 is a candidate Wnt-regulated MAPK transcriptional repressor. (**A**) Heatmap of scaled differential gene expression values (*z* scores) for 79 senescence-associated genes (SenMayo gene set) (36) in response to Wnt inhibition with or without *EPHA2* KO, *n* = 4–5 mice/group; treated with 37.5 mg/kg bid ETC-159 or vehicle for 56 hours. Loss of EPHA2 alone does not significantly alter the expression of senescence-associated genes despite MAPK activation. (**B**) Expression of representative senescence-associated genes selected from **A**. Despite activation of ERK, *EPHA2* KO alone does not induce these targets. Data represent mean ± SD; TPM, transcripts per million (*n* = 4–5 mice/group). Benjamini-Hochberg–adjusted *P* values are shown (adjusted **P < 0.01 and ****P < 0.0001). (**C**) Despite activation of ERK, loss of EPHA2 does not induce senescence in orthotopic xenografts. OCT tumor sections were stained for senescence-associated β-gal activity. Representative images are shown. Scale bars: 100 μm. (**D**) Wnt inhibition (56 hours) decreases *GATA3* gene expression in both parental and *EPHA2*-KO tumors. Benjamini-Hochberg–adjusted *P* values are shown (adjusted ****P < 0.0001). (**E**) Wnt inhibition (56 hours) decreases GATA3 protein abundance in both parental and *EPHA2*-KO tumors (2 independent *EPHA2*-KO clones). (**F**) In vitro knockdown of *GATA3* derepresses a subset of MAPK target genes in *EPHA2*-KO cells, as assessed by RT-qPCR 48 hours after knockdown. (**G**) Western blot shows reduced GATA3 protein abundance in *GATA3* siRNA–treated cells. (**H**) Western blot shows reduced GATA3 protein abundance in *GATA3* partial KO clone 15. (**I**) Moderate increase in expression of select MAPK target genes in *GATA3* partial KO clone 15 compared to parental HPAF-II cells cultured in vitro.

**Figure 5:**
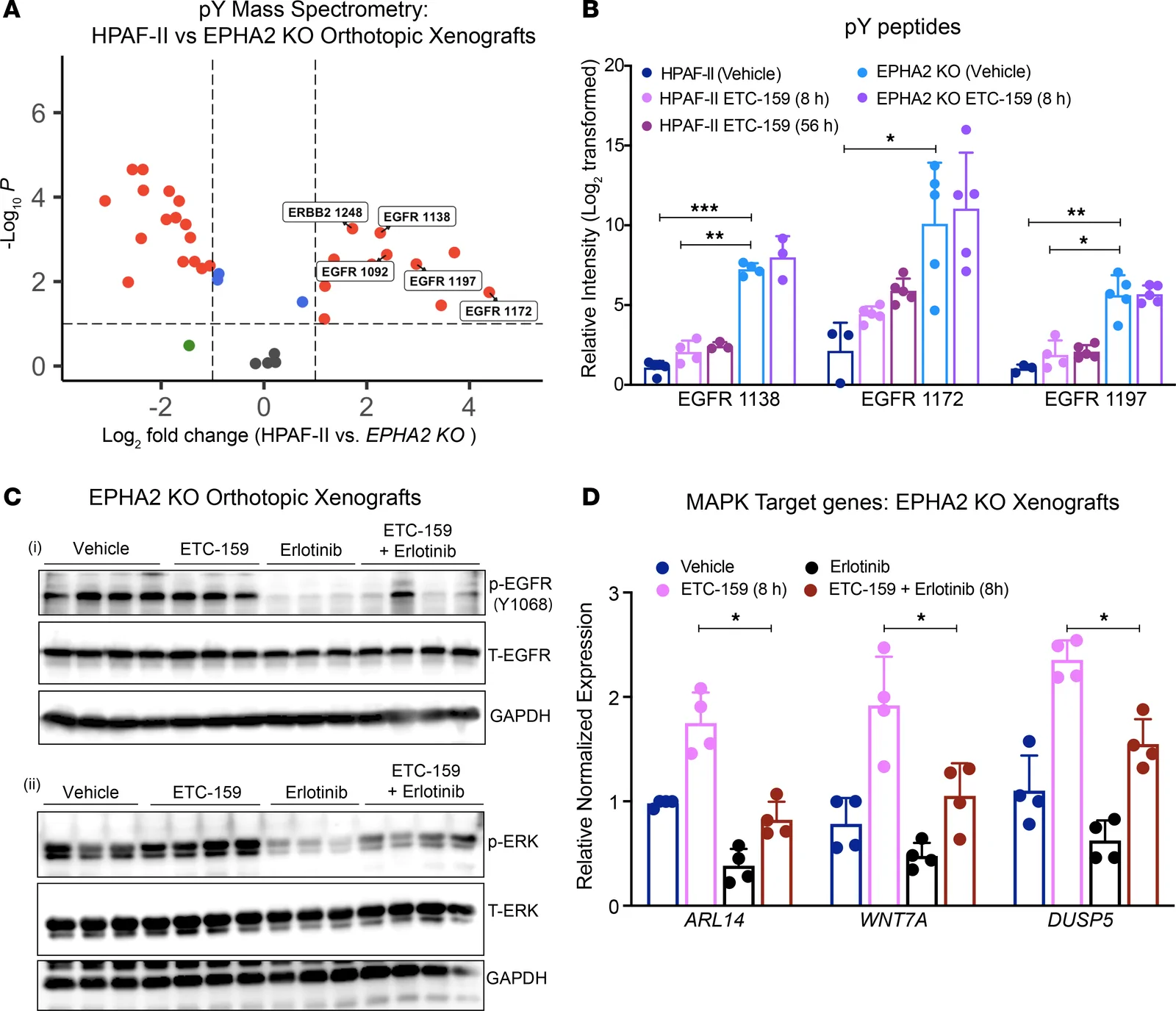
EGFR activation in response to *EPHA2* KO drives ERK phosphorylation and contributes to MAPK target gene expression after Wnt inhibition. (**A**) EPHA2 loss leads to phosphorylation of EGFR at specific tyrosine residues. Volcano plot representing RTKs phosphorylated in response to *EPHA2* KO in HPAF-II orthotopic tumors. Phosphoproteomic data obtained as described in Methods. (**B**) *EPHA2* KO is a more potent inducer of EGFR phosphorylation than Wnt inhibition. Specific phosphorylation sites are indicated. Only experimentally obtained datapoints are shown; imputed values were excluded. *n* = 3–5 mice/group. Differential analysis was performed using the R package limma with Benjamini-Hochberg adjustment for multiple testing (adjusted *P < 0.05, **P < 0.01, ***P < 0.001). (**C**) *EPHA2* KO–induced phosphorylation of EGFR (top) and ERK (bottom) is largely abrogated by treatment with erlotinib (100 mg/kg), an EGFR inhibitor. Mice with orthotopic xenografts received a single dose of the indicated drugs including ETC-159 (75 mg/kg), and tumors were harvested 8 hours later. (**D**) MAPK target genes induced by Wnt inhibition in *EPHA2*-KO tumors are sensitive to cotreatment with erlotinib. Expression of selected MAPK target genes was assessed by RT-qPCR in samples from **C**. Data were analyzed using unpaired, 2-tailed Mann-Whitney *U* test on ΔCq values, *P < 0.05. Relative normalized expression is shown.

**Figure 6:**
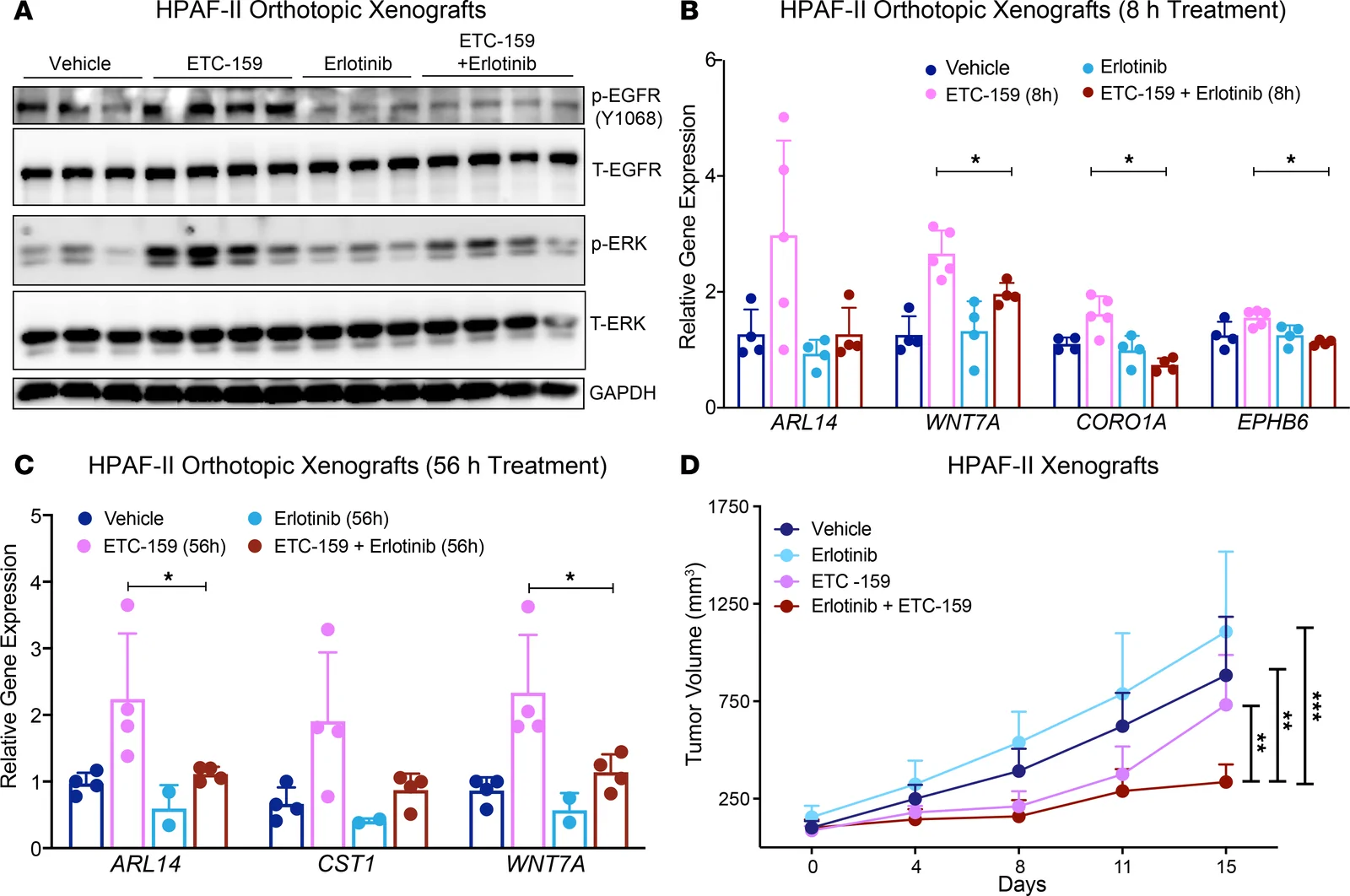
Wnt inhibition–mediated EGFR activation is partially responsible for ERK phosphorylation and MAPK target gene expression. (**A**) EGFR phosphorylation induced by Wnt inhibition is abrogated by erlotinib. Mice bearing HPAF-II orthotopic xenografts were treated with a single dose of ETC-159 (75 mg/kg) and/or erlotinib (100 mg/kg), and tumors were harvested 8 hours later. *n* = 3–4 mice/group. In the same samples, Wnt inhibition–induced ERK activation is also reduced by erlotinib. (**B** and **C**) Erlotinib treatment blunted the upregulation of MAPK target genes induced by ETC-159. (**B**) A single dose of 75 mg/kg 8 hours prior to harvest or (**C**) 37.5 mg/kg twice daily for 56 hours prior to harvest. Relative mRNA abundance of MAPK target genes was determined by RT-qPCR. *n* = 2–5 mice/group. Data were analyzed using unpaired, 2-tailed Mann-Whitney *U* test on ΔCq values, *P < 0.05. Relative normalized expression is shown. (**D**) ETC-159 and erlotinib in combination inhibit the growth of HPAF-II subcutaneous xenografts. Erlotinib alone (50 mg/kg/day) does not significantly inhibit tumor growth, while low-dose ETC-159 (10 mg/kg once daily) slows tumor progression. Combining ETC-159 with erlotinib markedly reduced tumor growth. *n* = 6–10 tumors/group. Tumor volumes on day 15 were analyzed by unpaired, 2-tailed Mann-Whitney *U* tests, **P < 0.01, ***P < 0.001. Data are presented as mean ± SD.

**Figure 7:**
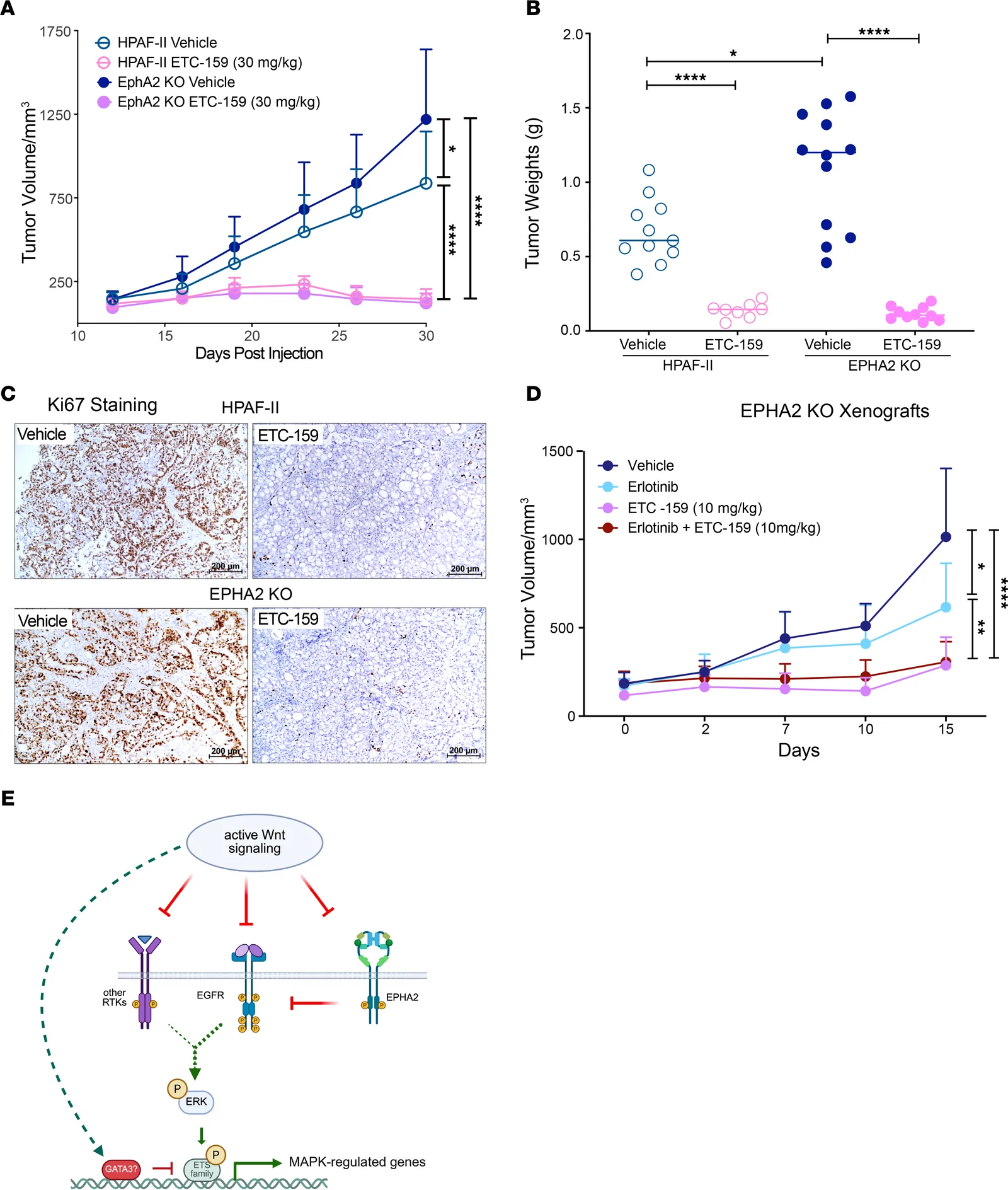
*EPHA2* KO increases sensitivity to Wnt and EGFR inhibition. (**A** and **B**) KO of *EPHA2* potentiates the growth of HPAF-II subcutaneous xenografts (*P* < 0.05 at postinjection day 30). *EPHA2*-KO xenografts are significantly larger than their WT counterparts. *EPHA2*-KO tumors retain their sensitivity to Wnt inhibition. *n* = 8–12 subcutaneous tumors/group, treated with ETC-159 30 mg/kg/d or vehicle for 20 days. Tumor volumes on day 30 and tumor weights were analyzed by unpaired 2-tailed Mann-Whitney *U* test, ****P < 0.0001. Data are presented as mean ± SD. (**C**) Parental and *EPHA2*-KO HPAF-II xenografts have comparable numbers of Ki67-positive proliferating cells. *EPHA2*-KO xenografts have reduced proliferation in response to Wnt inhibition, similar to the effect of Wnt inhibition on control xenografts. Scale bars: 200 μm. (**D**) *EPHA2* KO increases tumor sensitivity to both low-dose ETC-159 (10 mg/kg) and erlotinib (50 mg/kg) treatment. *n* = 6–10 subcutaneous tumors/group. Tumor volumes at day 15 were analyzed by unpaired 2-tailed Mann-Whitney *U* test, *P < 0.05, **P < 0.01, ****P < 0.0001. Data are presented as mean ± SD. (**E**) Model: Active Wnt signaling slows receptor recycling, thereby preventing the activation of RTKs such as EPHA2 and EGFR. It also stabilizes a repressive transcription factor (e.g., GATA3). When Wnt signaling is inhibited (e.g., via ETC-159 or *TCF7L2* KO), EGFR becomes activated, ERK is phosphorylated, and concurrently the repressive factor(s) is deactivated, turning on MAPK target gene expression. However, when EPHA2 alone is blocked, EGFR and ERK are still phosphorylated but the Wnt-regulated repressor remains active, preventing activation of MAPK target genes.

## Version 1 09/08/2026

In-Press Preview

## Version 2 10/08/2026

Electronic publication
