# Supplementary material for: EPHA2 and WNT reciprocally regulate MAPK-dependent gene expression in a pancreatic cancer model
Source: JCI Insight. 2026 Sep 8;11(19):e202630. doi: 10.1172/jci.insight.202630 (PMC13726417; doi:10.1172/jci.insight.202630)
Supplement: Supplementary file 1 [file jciinsight-11-202630-s079.pdf]

Figure S1

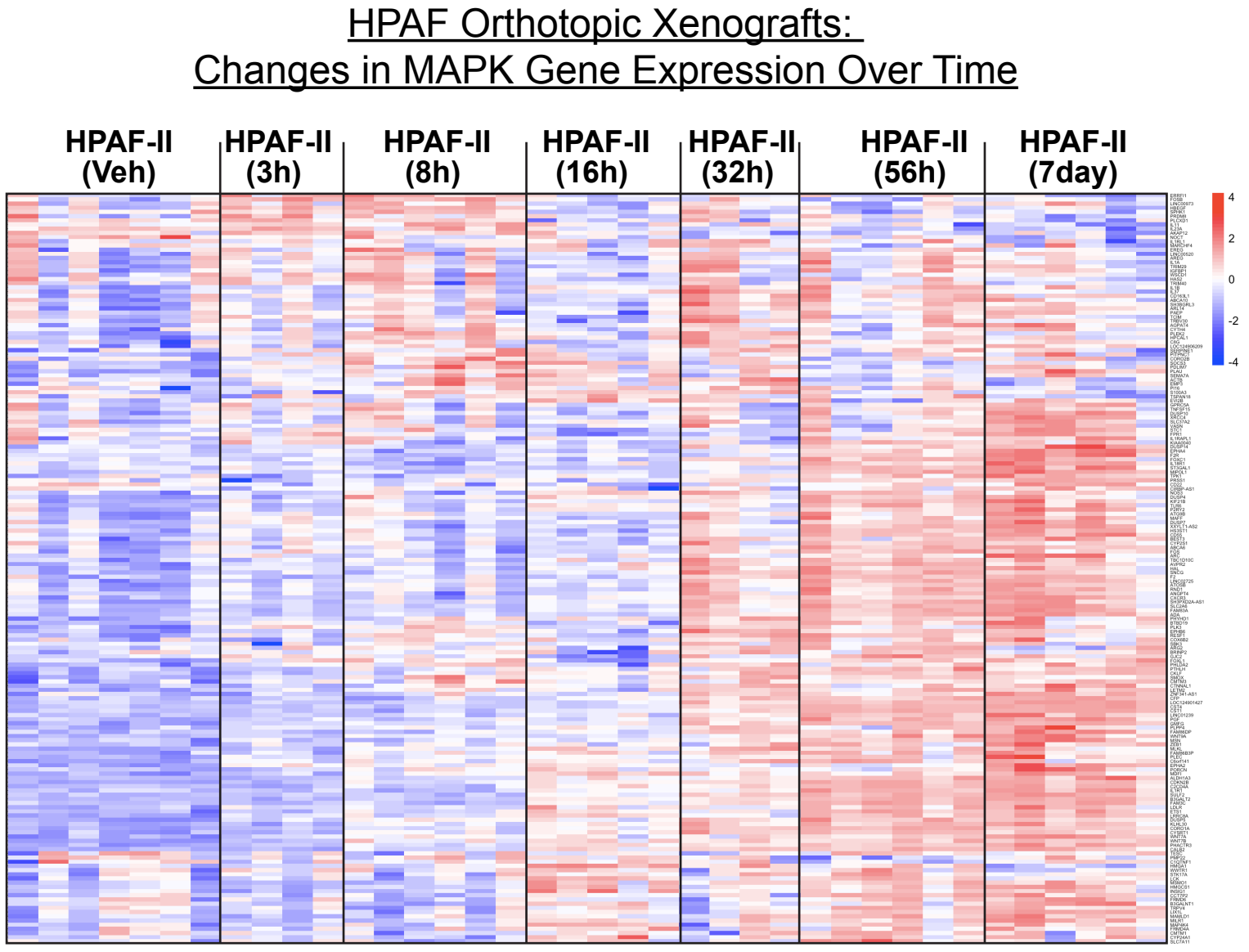

Figure S1: Wnt inhibition upregulates MAPK target gene expression. Expression levels of bona fide MAPK target genes in HPAF-II orthotopic tumors were assessed by RNA-seq (n = 4-5 mice per group). Heatmap shows z-scores for 179 MAPK target genes following Wnt inhibition at 3, 8, 16, 32, 56 and 168 hours (7 days).

Figure S2:

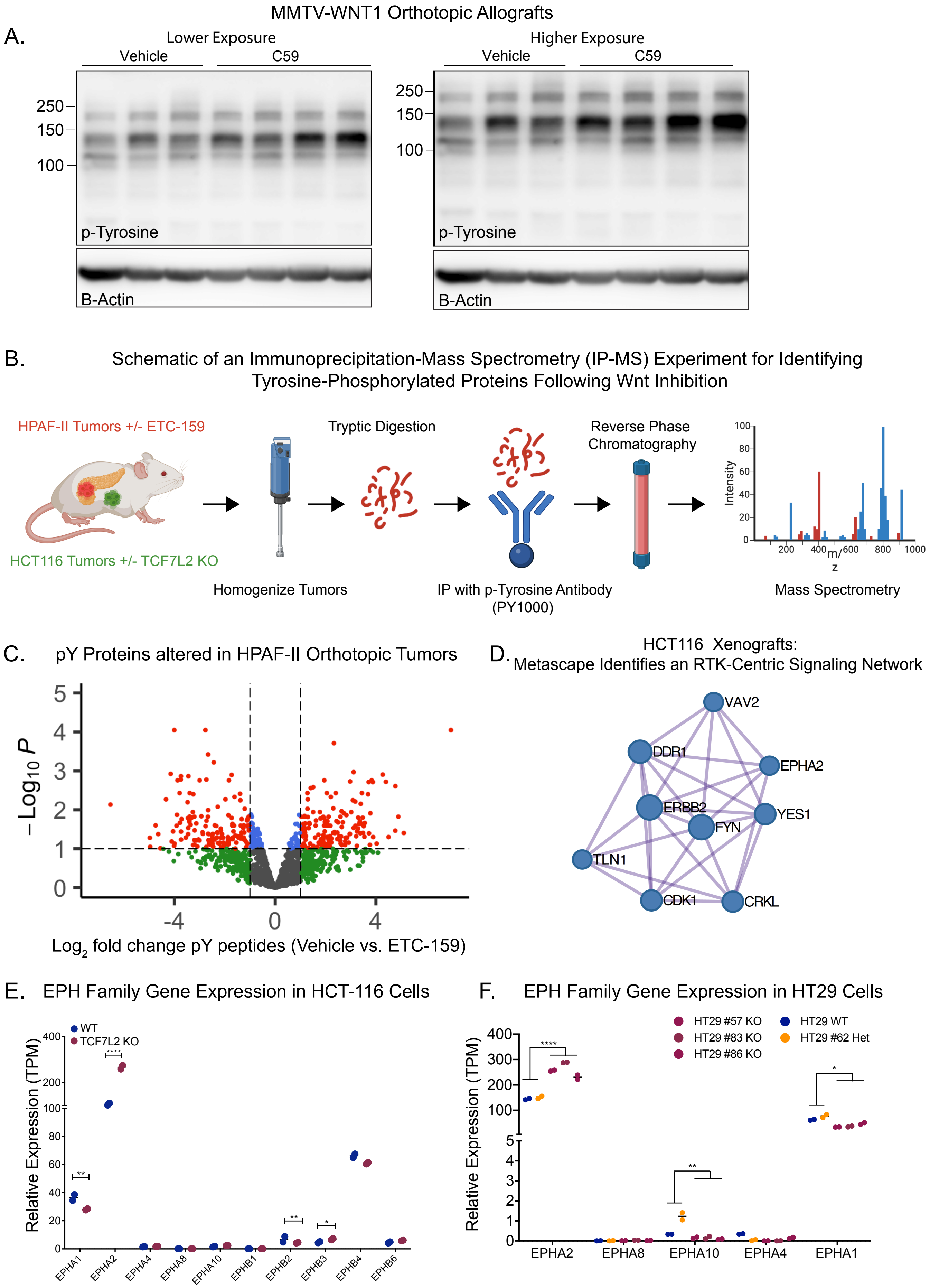

Figure S2: pY proteins altered by Wnt inhibition (A) A structurally distinct PORCN inhibitor induces tyrosine phosphorylation in Wnt-driven MMTV-Wnt1 mammary tumor allografts. (B) Schematic of the immunoprecipitation mass spectrometry (IP-MS) workflow used to identify tyrosine-phosphorylated proteins. (C) Volcano plot showing changes in tyrosine-phosphorylated proteins after 56 hours of Wnt inhibition. (D) A phosphotyrosine protein interaction network centered on EPHA2 is detected by IP-MS in HCT116 subcutaneous xenografts. (E-F) EPHA2 is the most highly expressed EphA receptor in HCT116 and HT29 cells; EPHB6 is kinase-deficient. Benjamini–Hochberg adjusted p-values are shown with ****padj <0.0001.

Figure S3

A. Schematic of a two-guide CRISPR KO strategy targeting EphA2 exons 10–15

EPHA2 (ENSG00000142627) (31,751 base pairs)

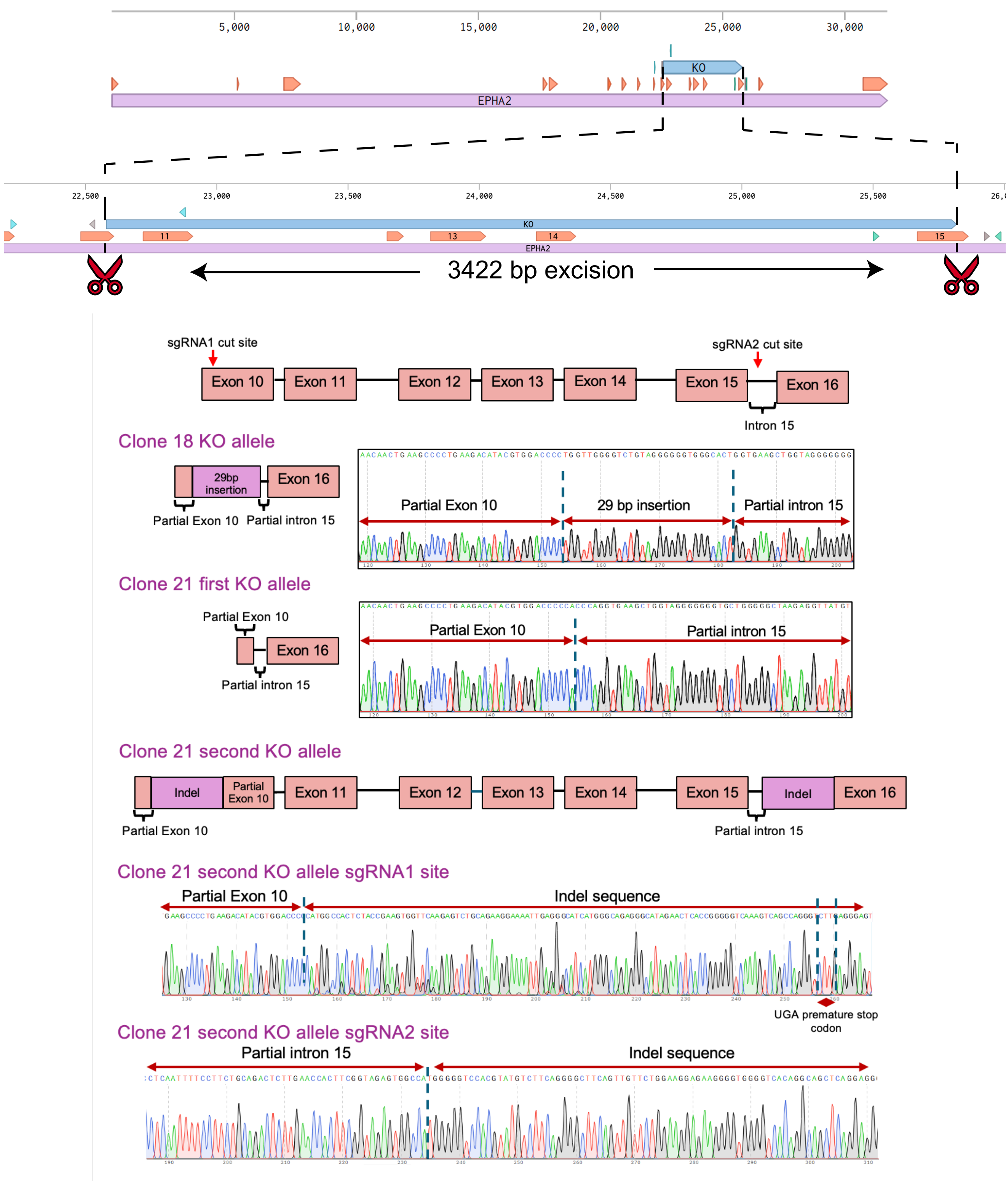

B.

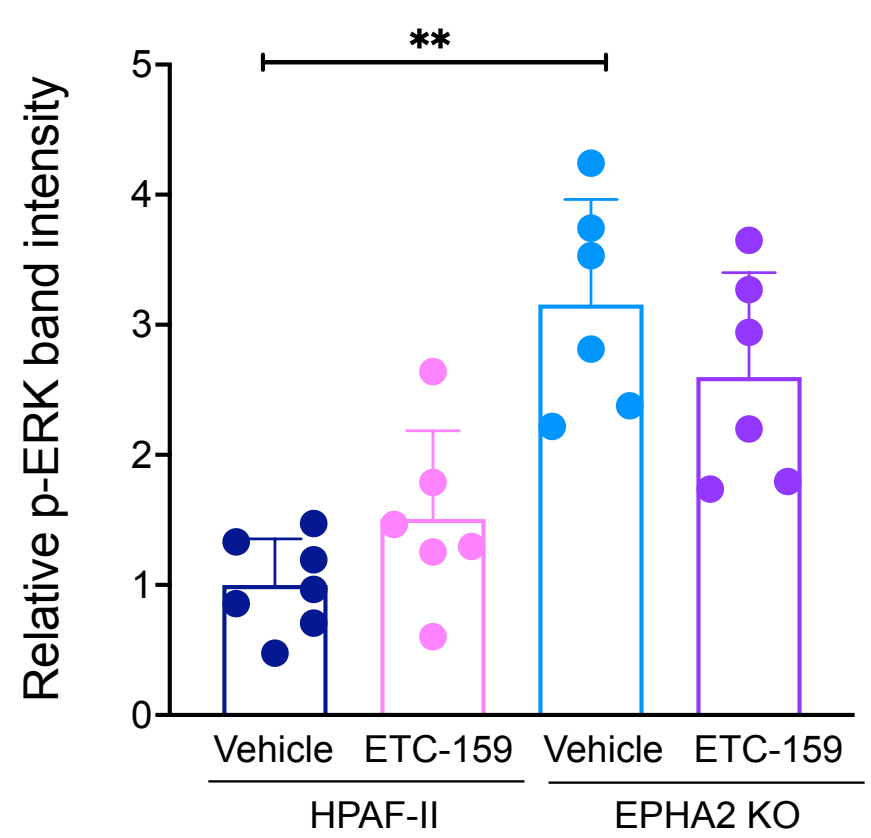

C.

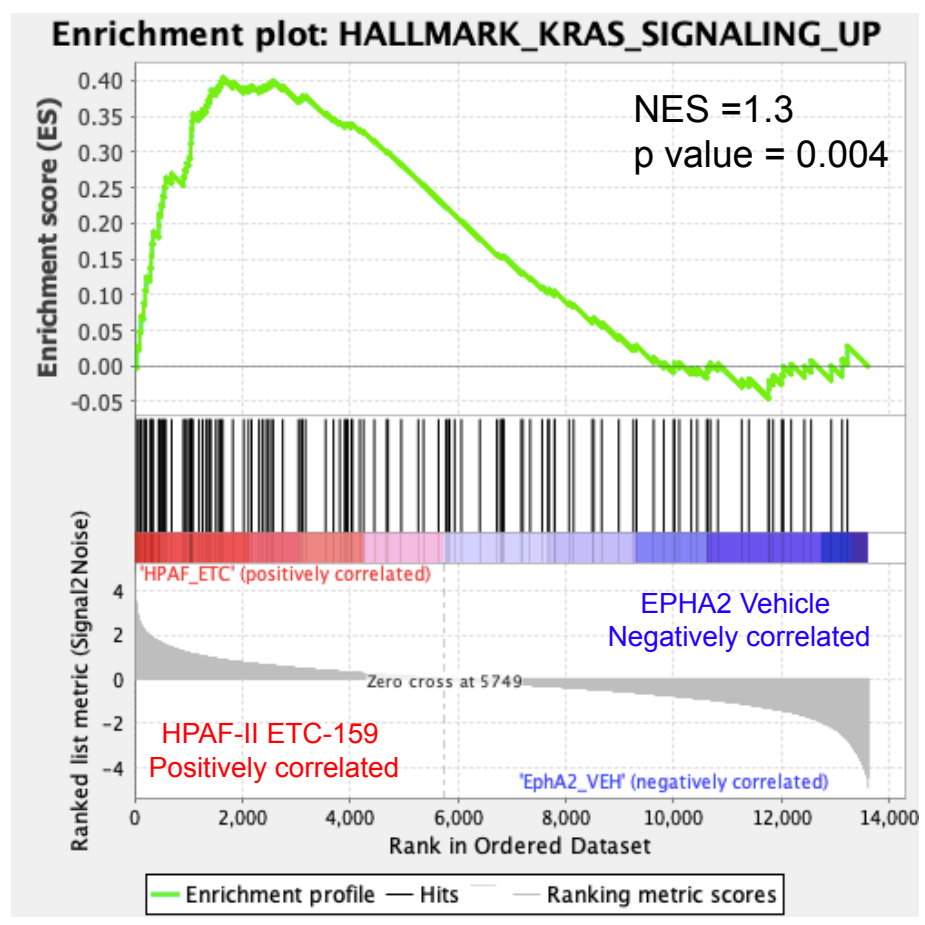

Figure S3: *EPHA2* knockout induces ERK phosphorylation without concomitant upregulation of MAPK target genes.

(A) Schematic of the two-guide CRISPR KO strategy targeting EPHA2 and Sanger sequencing of EPHA2 KO clones #18 and #21.

(B) Quantification of p-ERK abundance in HPAF-II and EPHA2 KO orthotopic tumors from two independent Western blots. A representative blot is shown in Fig. 3B; additional tumors included in the quantification are not shown. n = 6–7 tumors per group.

(C) Despite elevated p-ERK levels, genes from the Hallmark KRAS signaling signature are not enriched in EPHA2 KO tumors but are enriched in ETC-159-treated parental HPAF-II tumors. NES (Normalized Enrichment Score) and nominal p values are indicated.

Figure S4

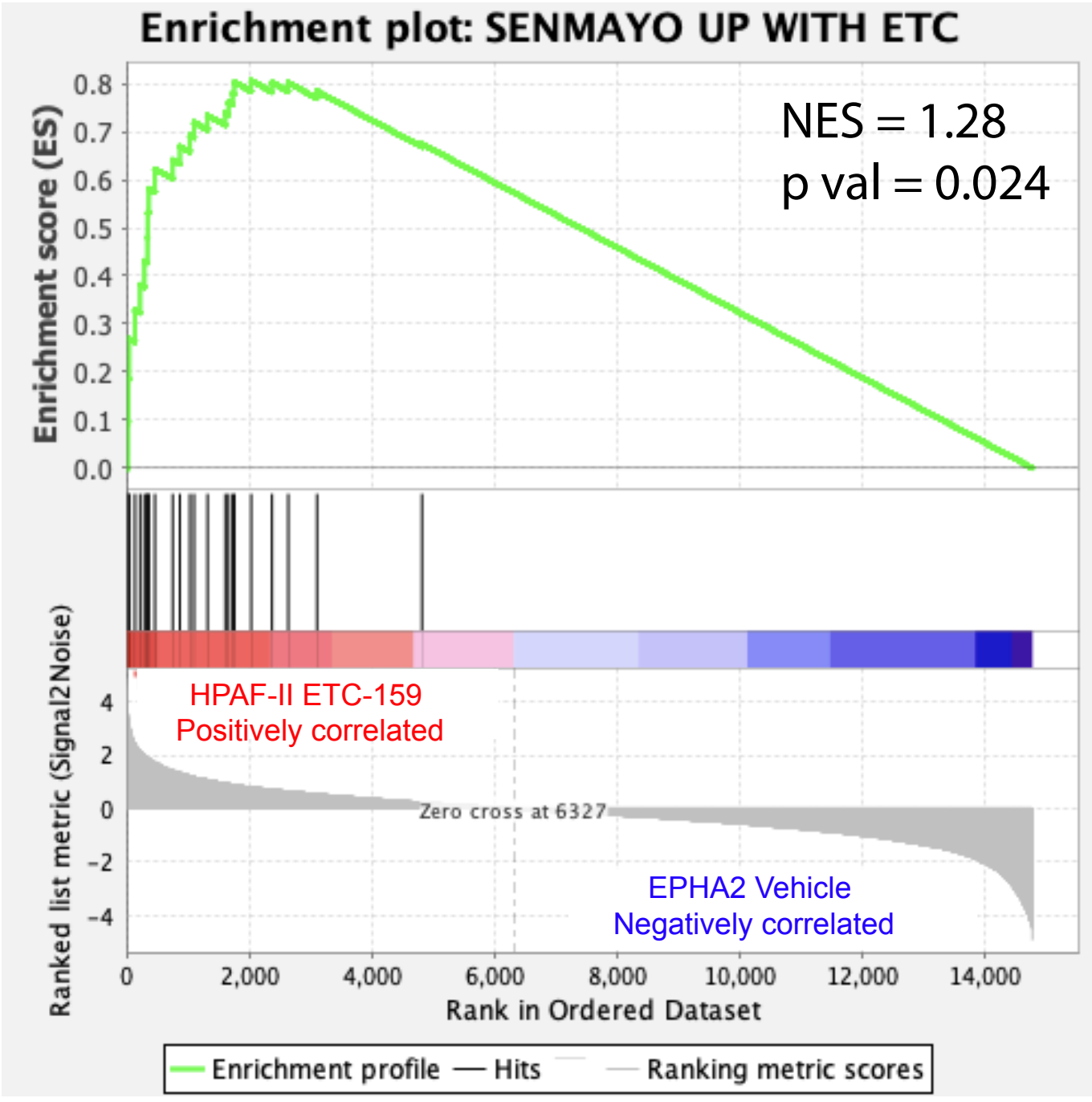

Figure S4: EPHA2 knockout does not increase expression of senescence-associated genes. GSEA reveals stronger enrichment of senescence-associated genes in ETC-159 treated WT tumors compared to EPHA2 KO tumors, despite both exhibiting high levels of p-ERK. NES = Normalized Enrichment Score and nominal p values are indicated.

Figure S5

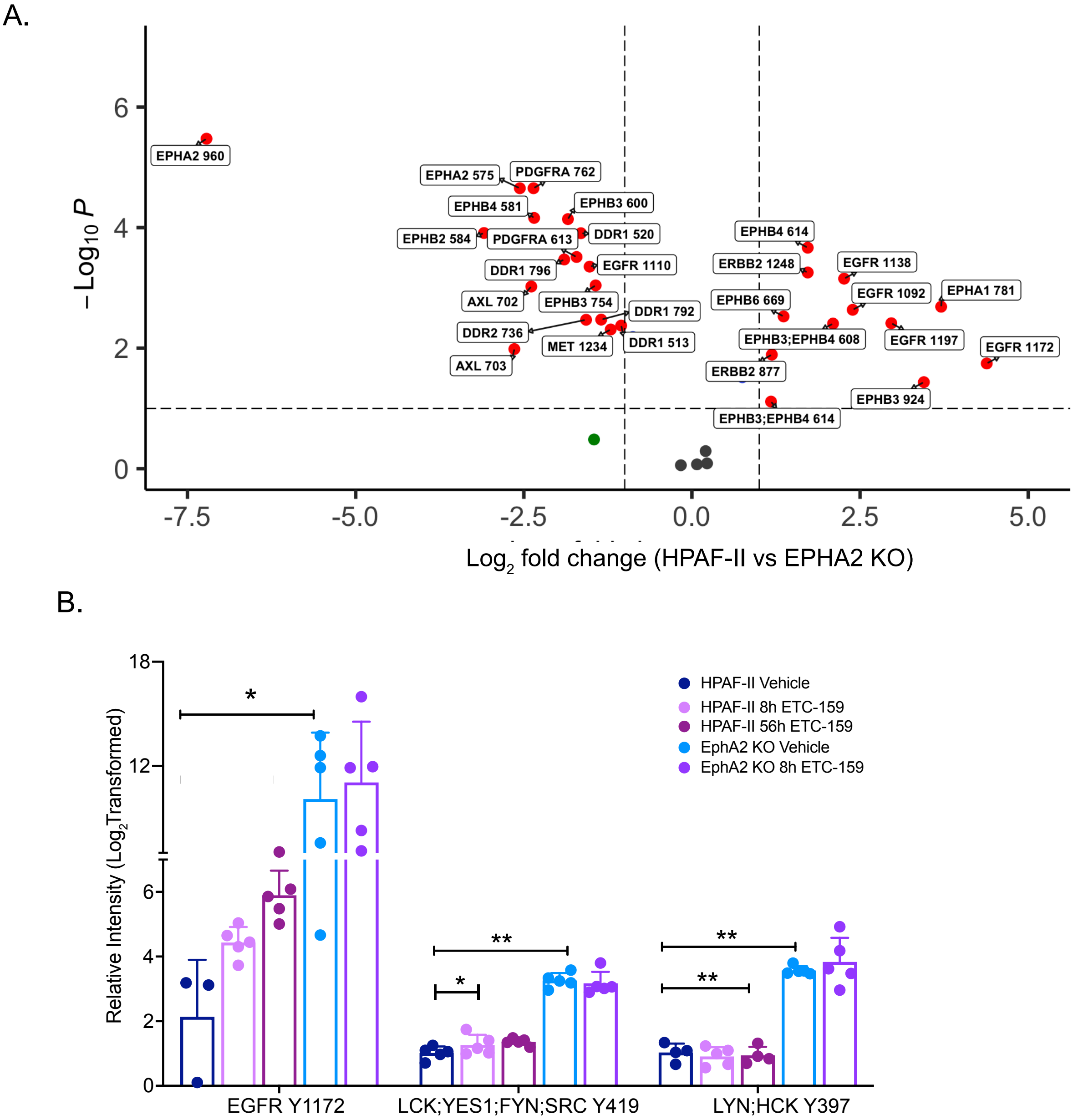

Figure S5: (A) EPHA2 loss leads to phosphorylation of EGFR at specific tyrosine residues. Volcano plot representing receptor tyrosine kinases (RTKs) phosphorylated in response to EPHA2 KO in HPAF-II orthotopic tumors. Phosphoproteomic data was obtained as described in Methods.

(B) EPHA2 KO is a more potent inducer of Src family kinases compared to Wnt inhibition. Specific phosphorylation sites are indicated. Only experimentally obtained datapoints are shown; imputed values were excluded. N = 3-5 mice per group. Data were analyzed using limma with Benjamini–Hochberg correction for multiple testing, * padj <0.05, ** padj <0.01, *** padj <0.001.

Figure S6

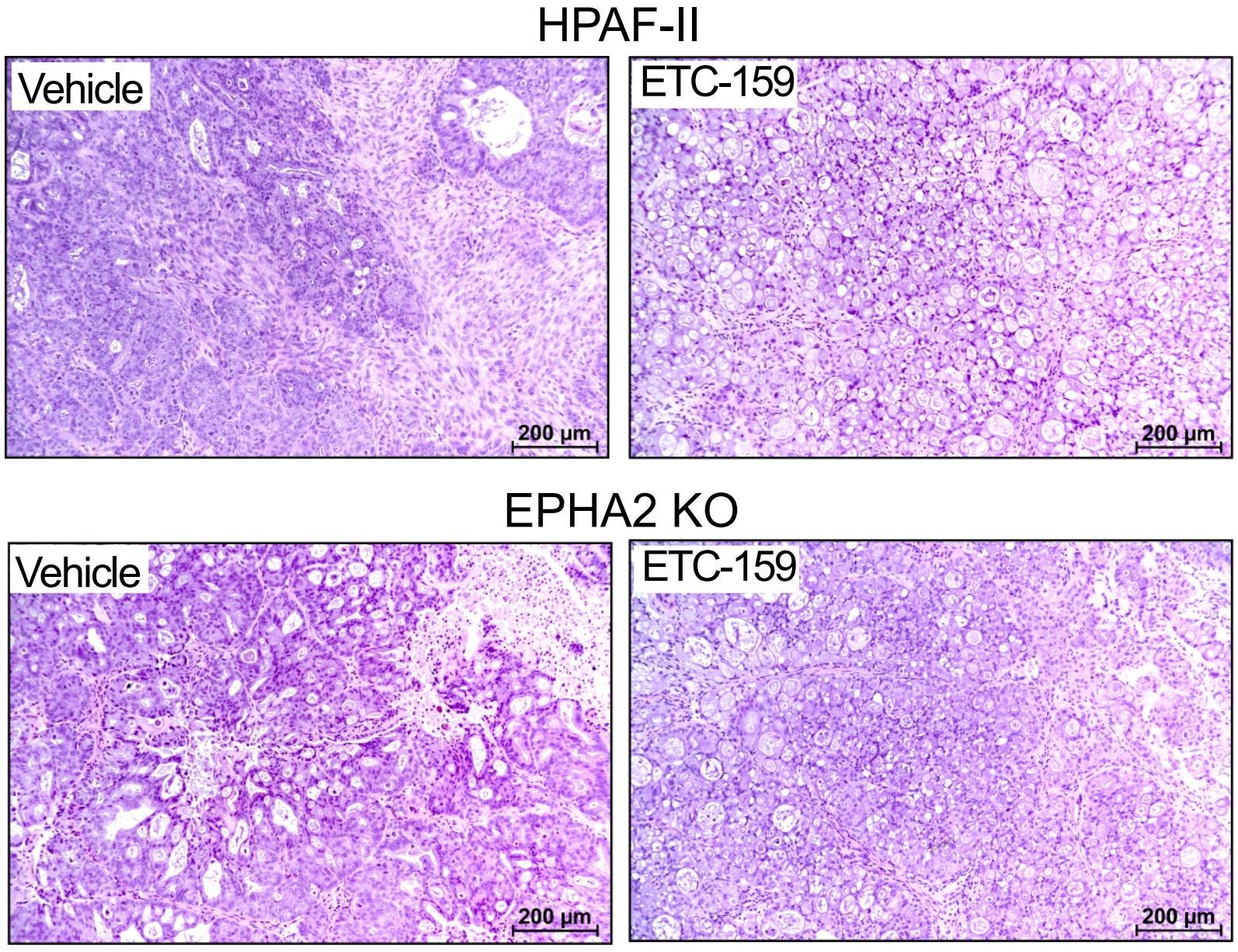

Fig S6:EPHA2 knockout preserves tumor morphology. EPHA2 knockout tumors (bottom) have a morphology similar to parental HPAF-II tumors (top) and remain sensitive to ETC-159 treatment. Representative H&E images shown.

Figure S7

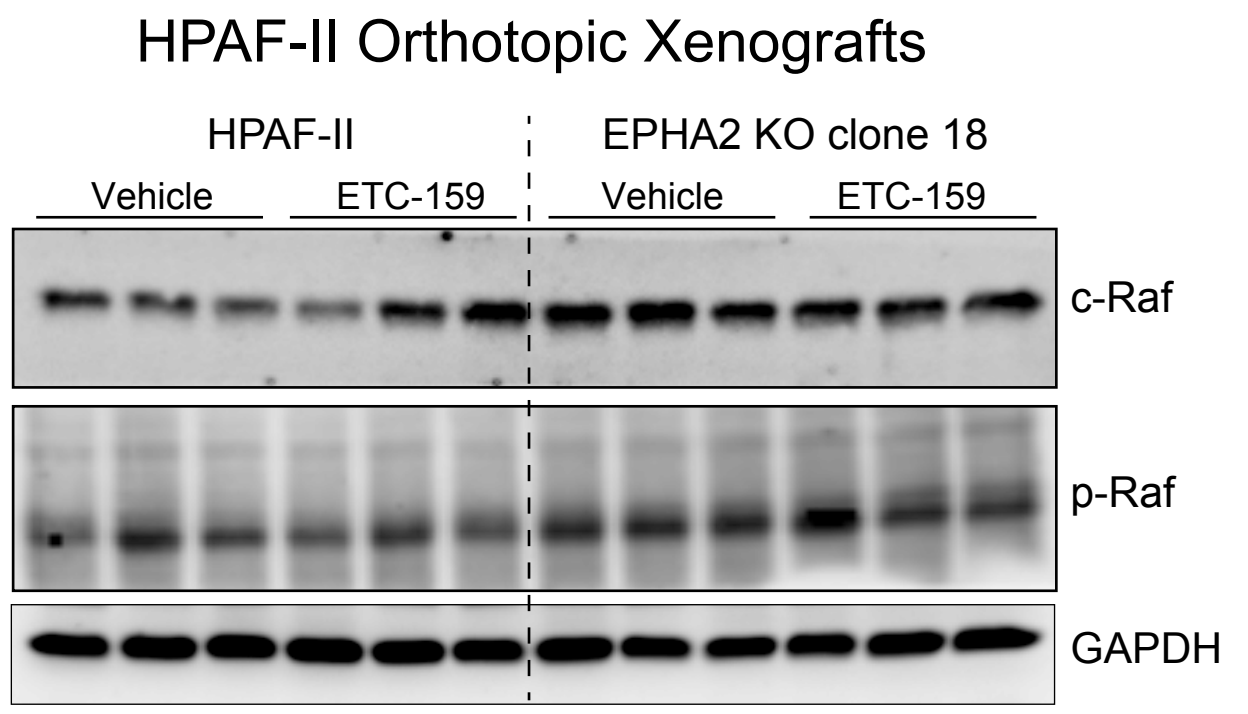

Figure S7: EPHA2 loss leads to an increase in total c-RAF abundance without a corresponding increase in c-RAF phosphorylation.
