# Supplementary material for: EPHA2 and WNT reciprocally regulate MAPK-dependent gene expression in a pancreatic cancer model
Source: JCI Insight. 2026 Sep 8;11(19):e202630. doi: 10.1172/jci.insight.202630 (PMC13726417; doi:10.1172/jci.insight.202630)
Supplement: Supplementary file 2 [file jciinsight-11-202630-s080.pdf]

Unedited WBs

1A,B

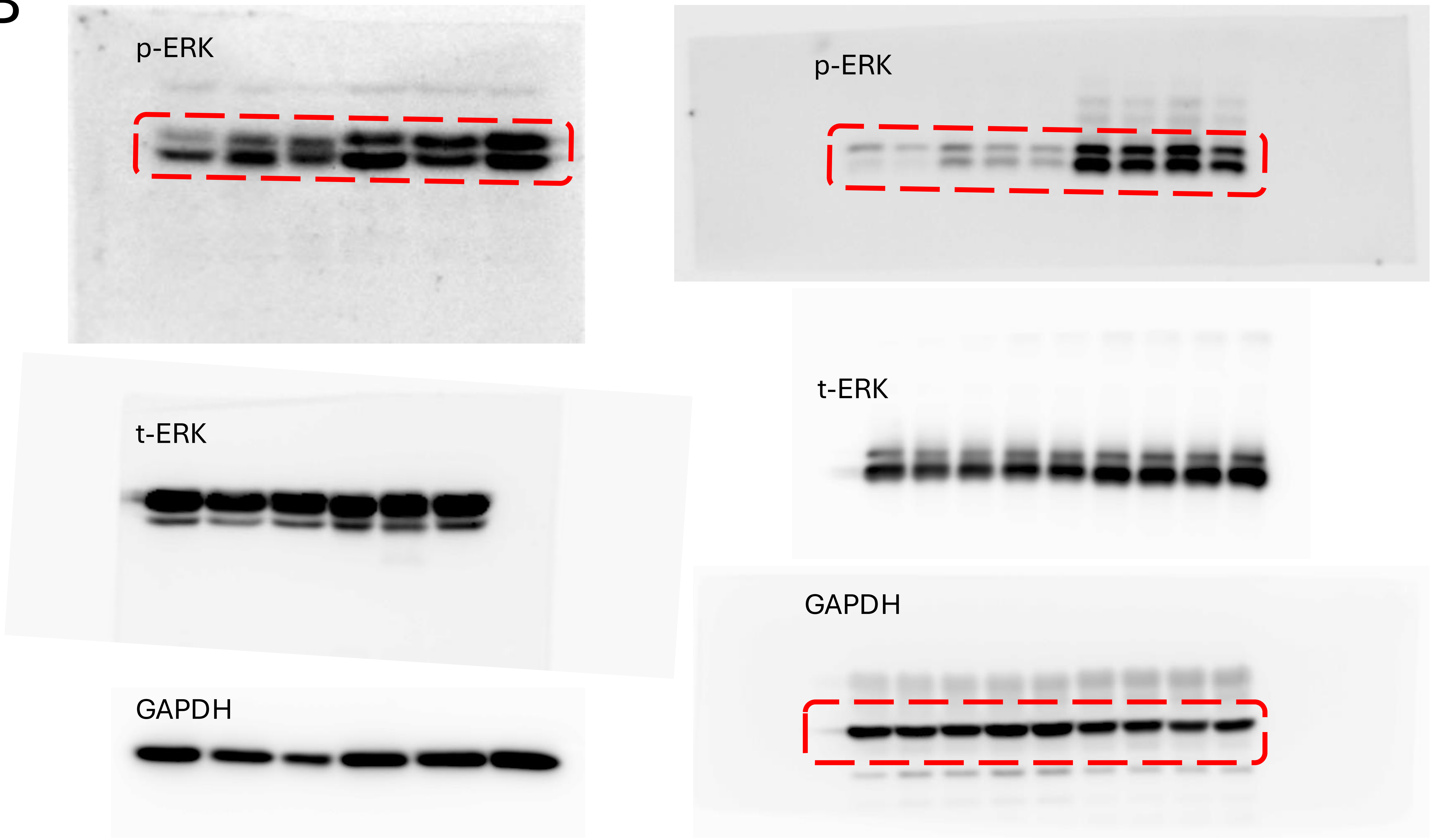

2A,B

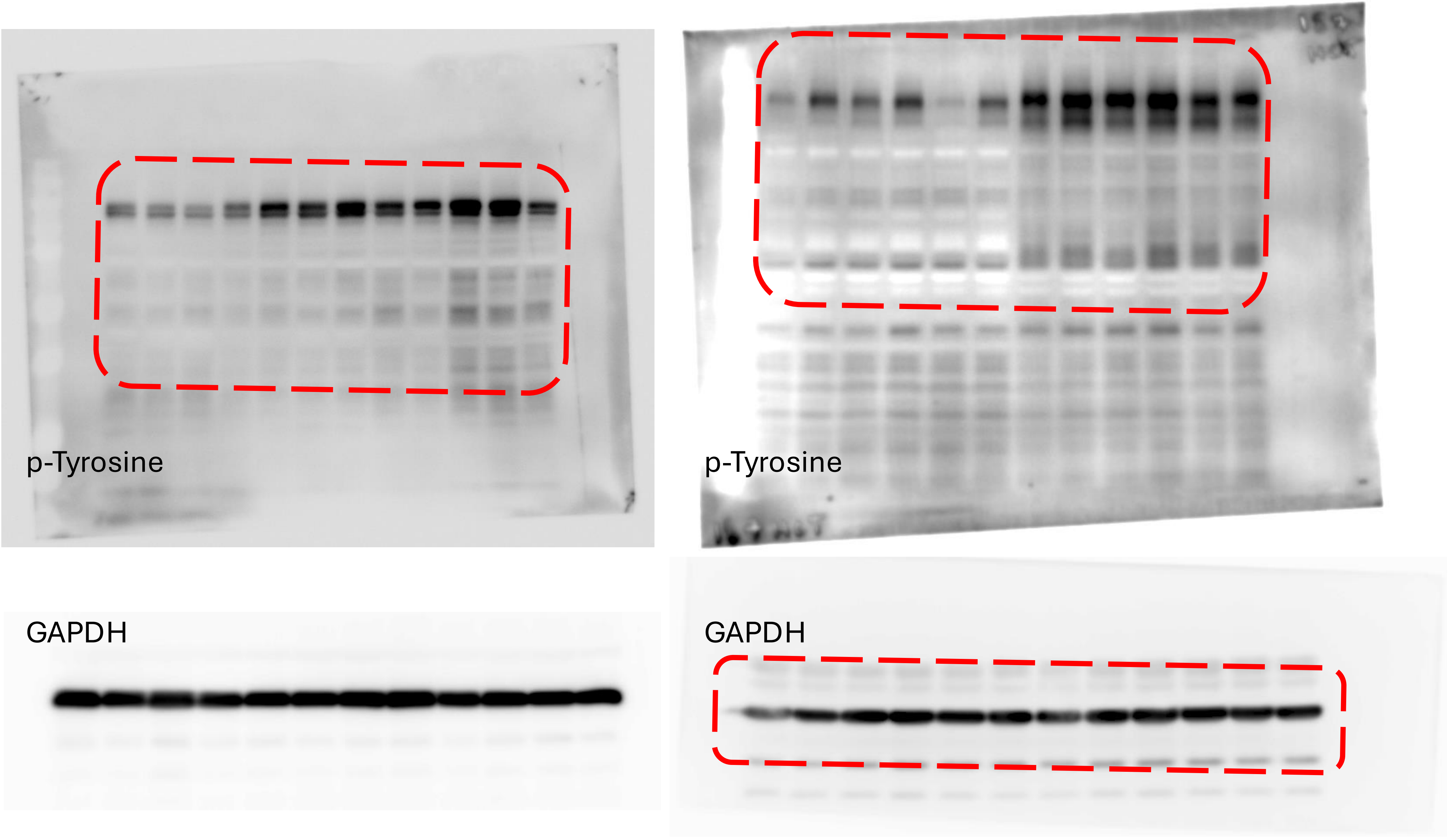

2E

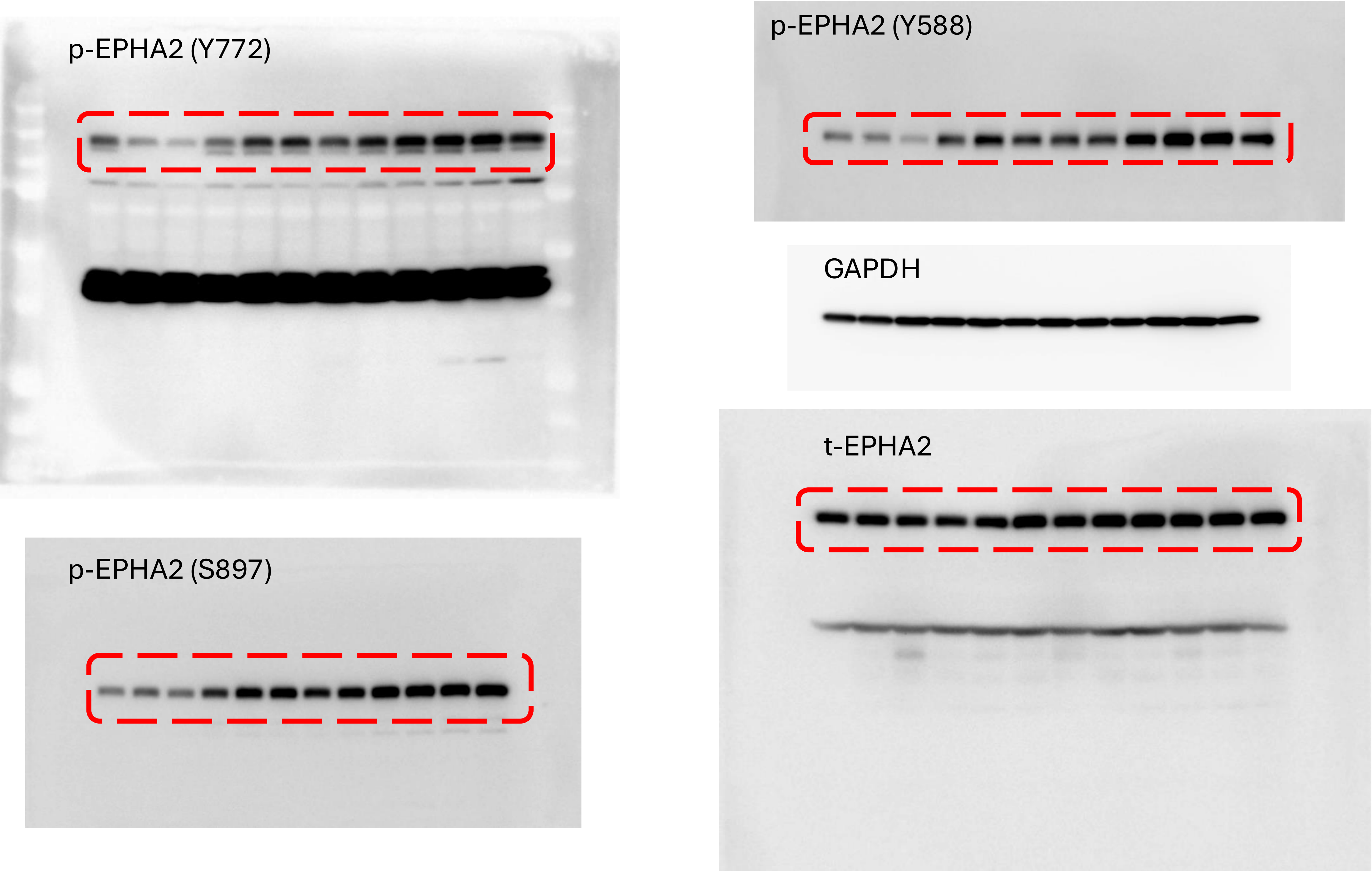

2F

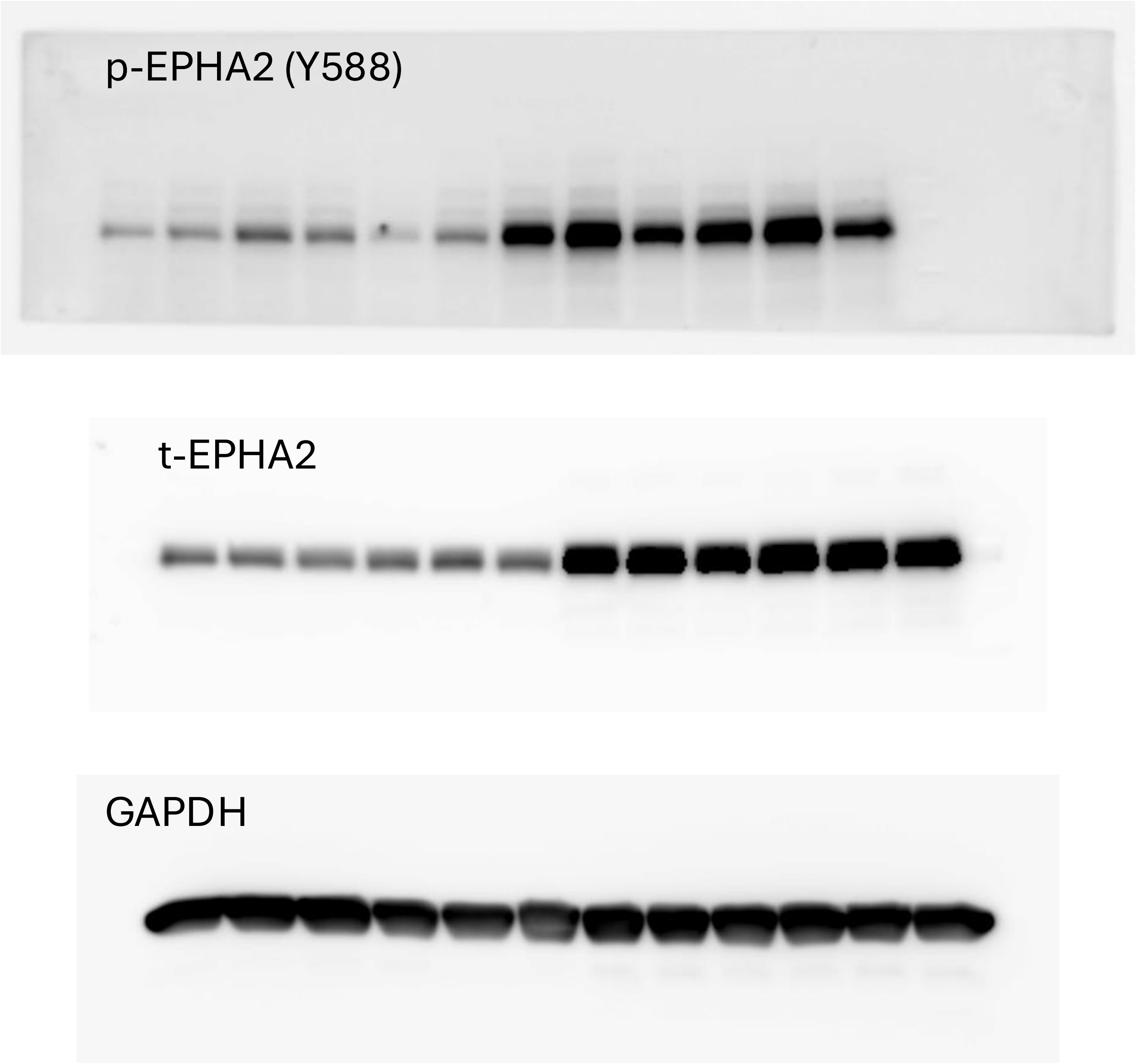

3A,B

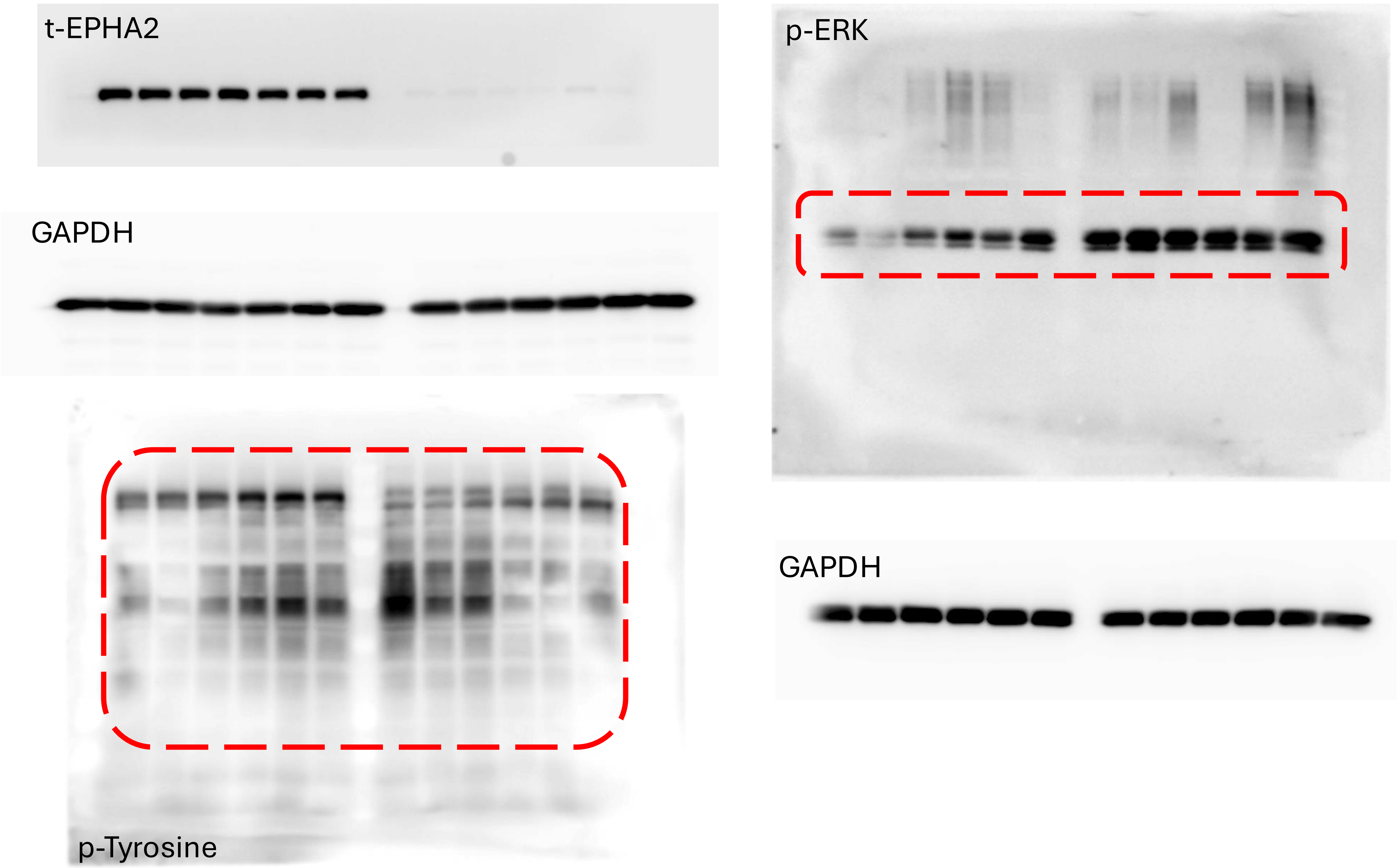

3C,D

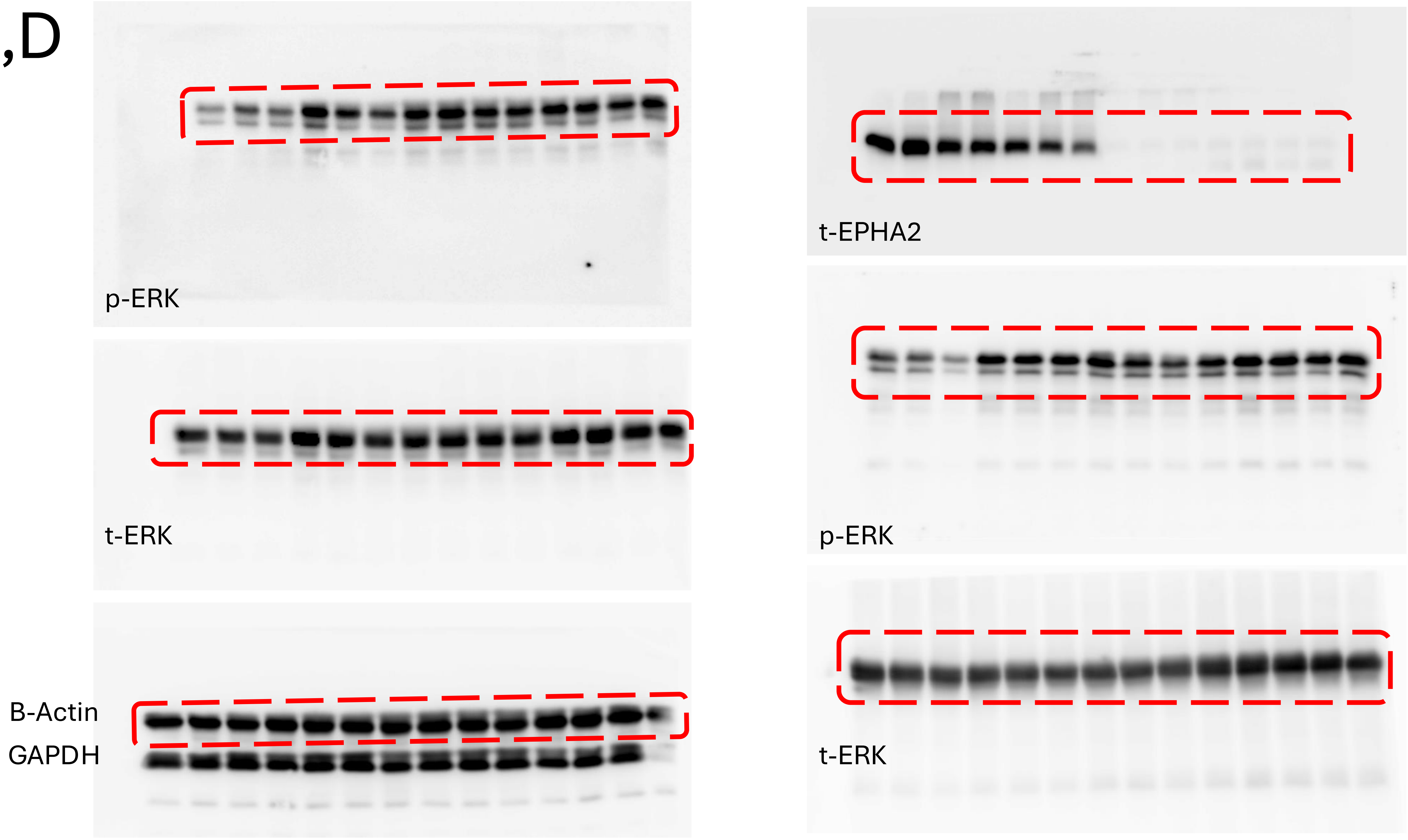

4E,G,H

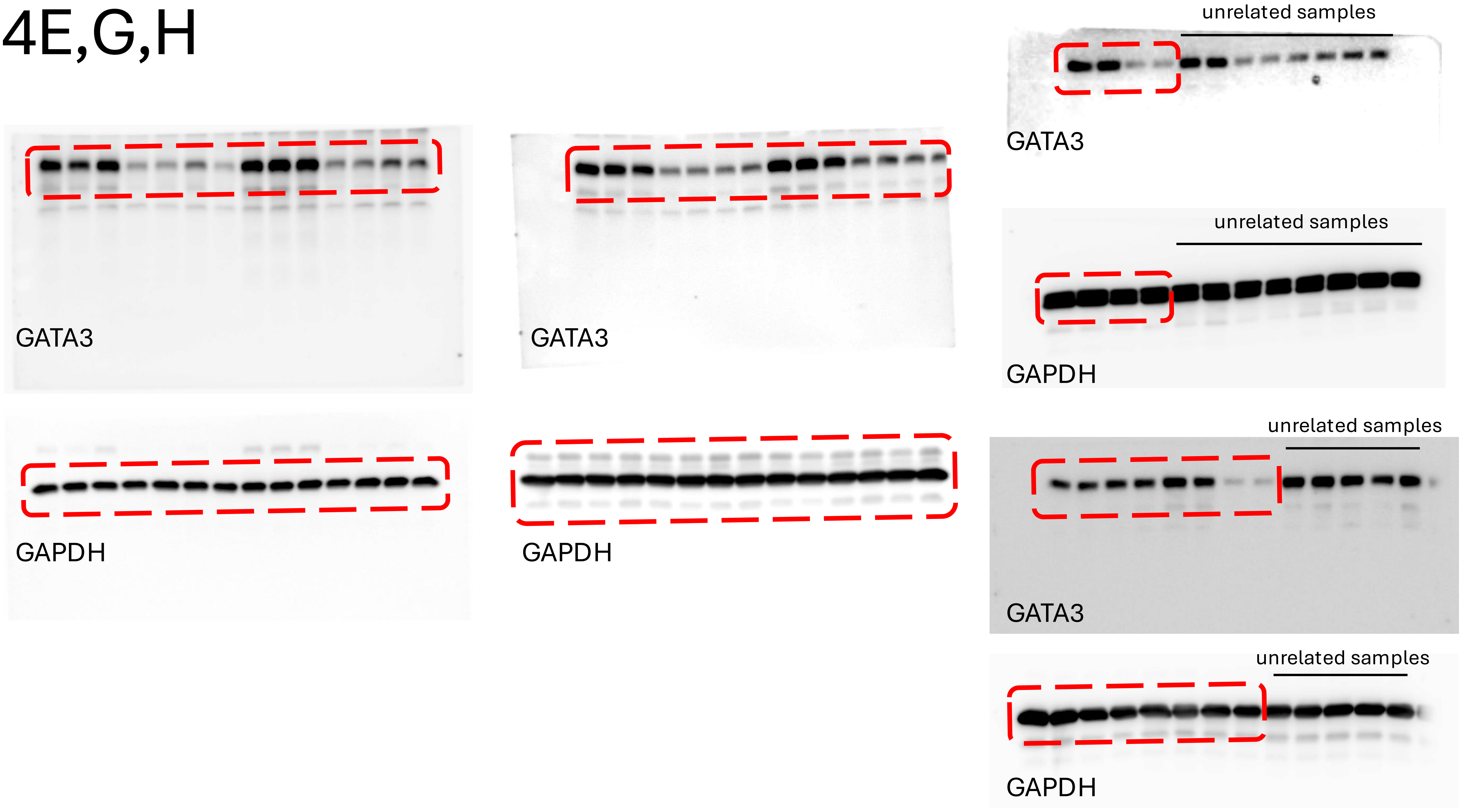
GATA3
GAPDH
GATA3
GAPDH
unrelated samples
GATA3
unrelated samples
GAPDH
unrelated samples
GATA3
unrelated samples
GAPDH

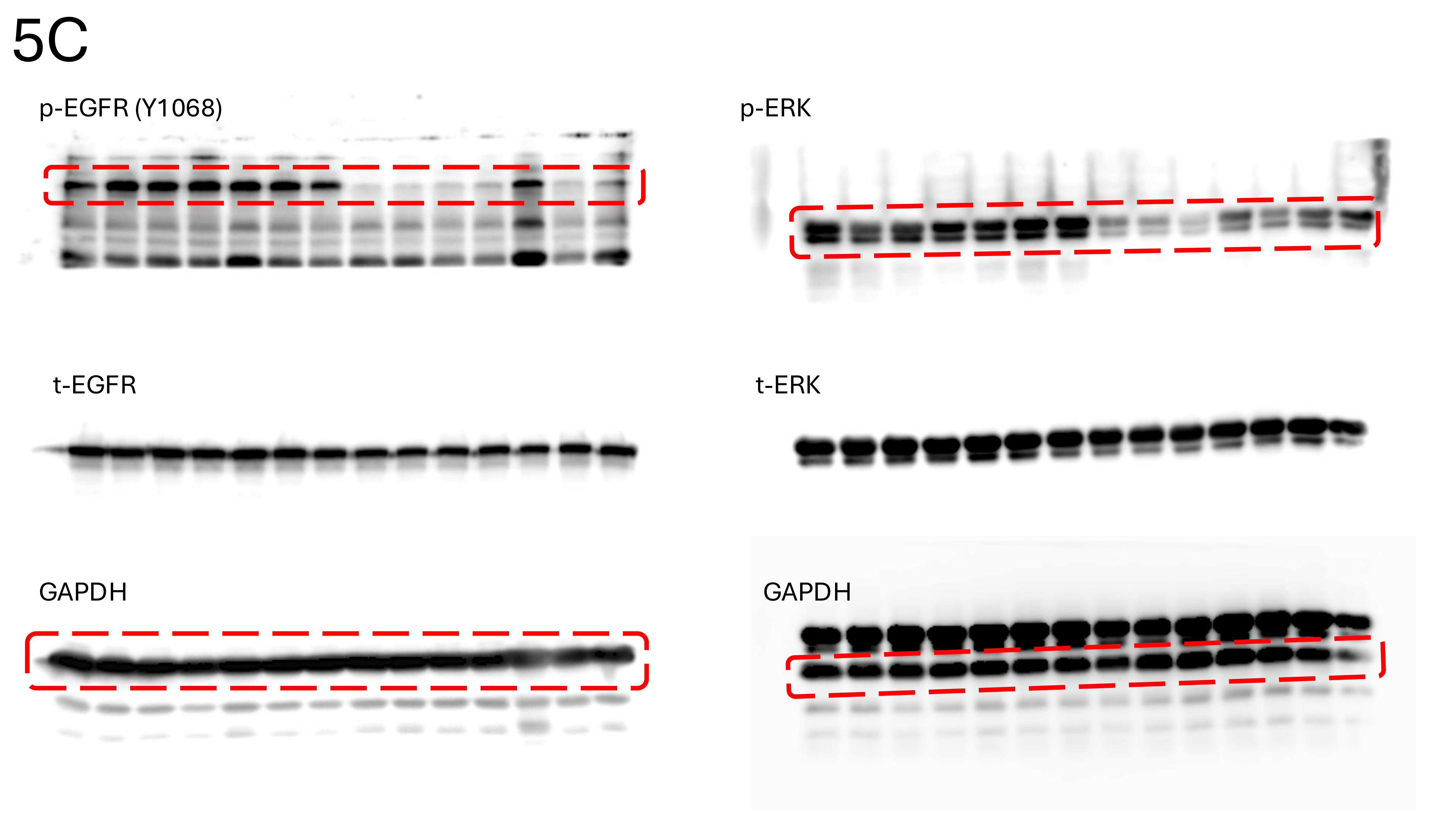
5C
p-EGFR (Y1068)
p-ERK
t-EGFR
t-ERK
GAPDH
GAPDH

6A,B

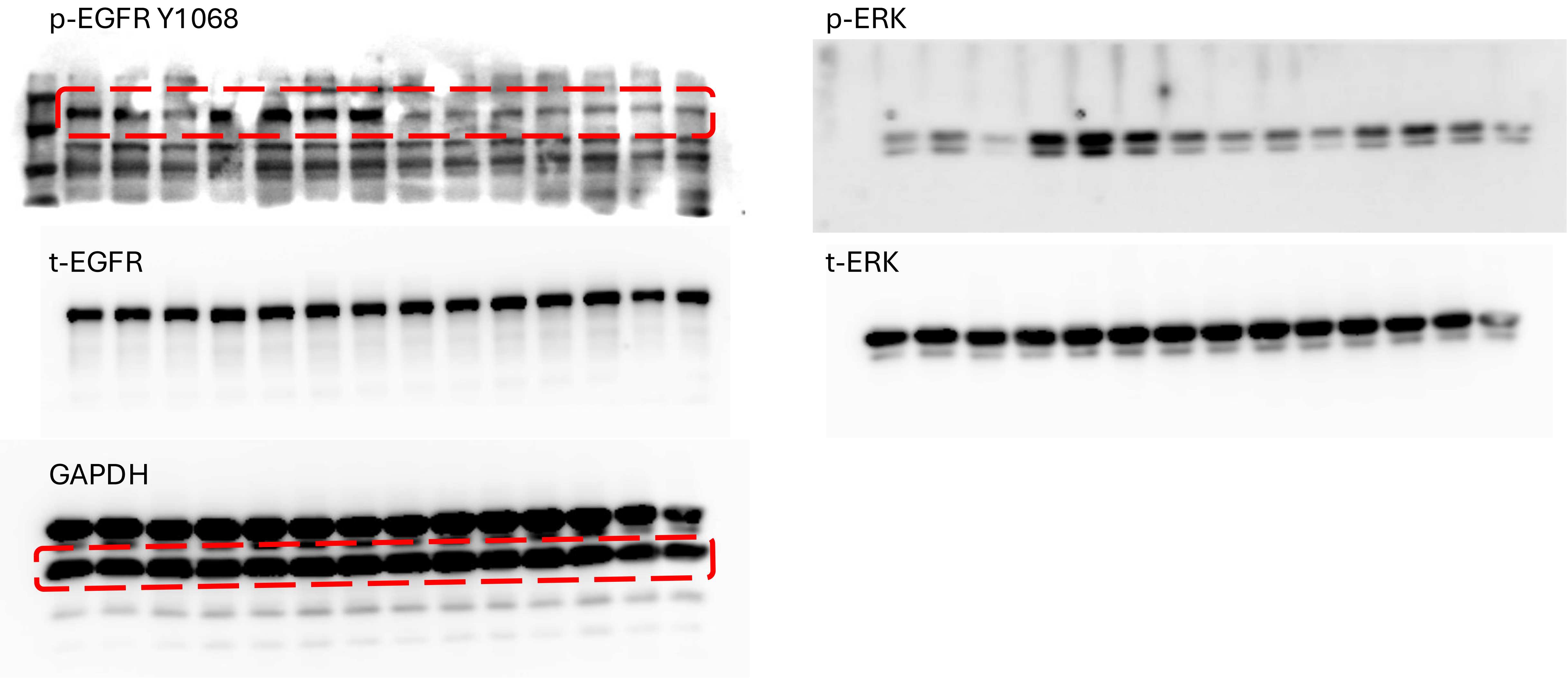

Note: A common loading control is shown for proteins analyzed on parallel gels loaded with the same lysates.

S2A

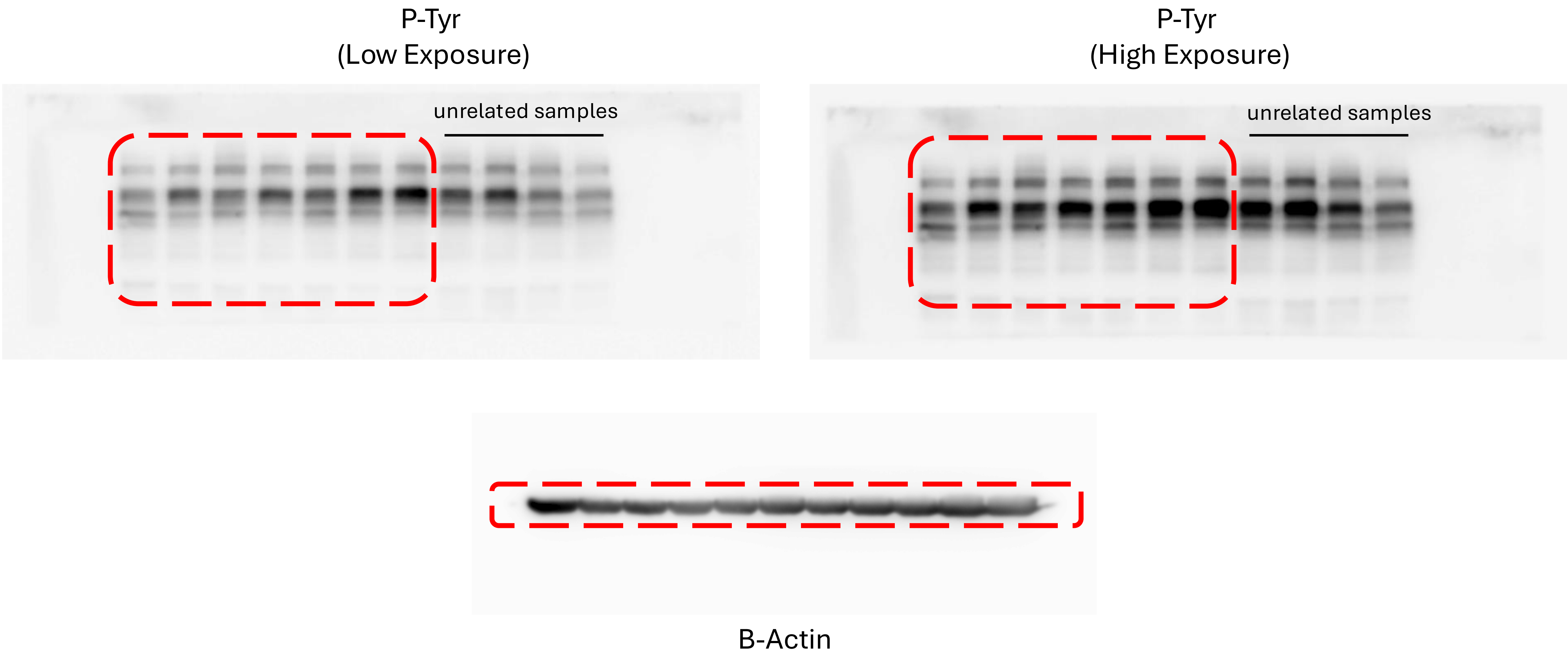
P-Tyr
(Low Exposure)
unrelated samples
P-Tyr
(High Exposure)
unrelated samples
B-Actin

S7A

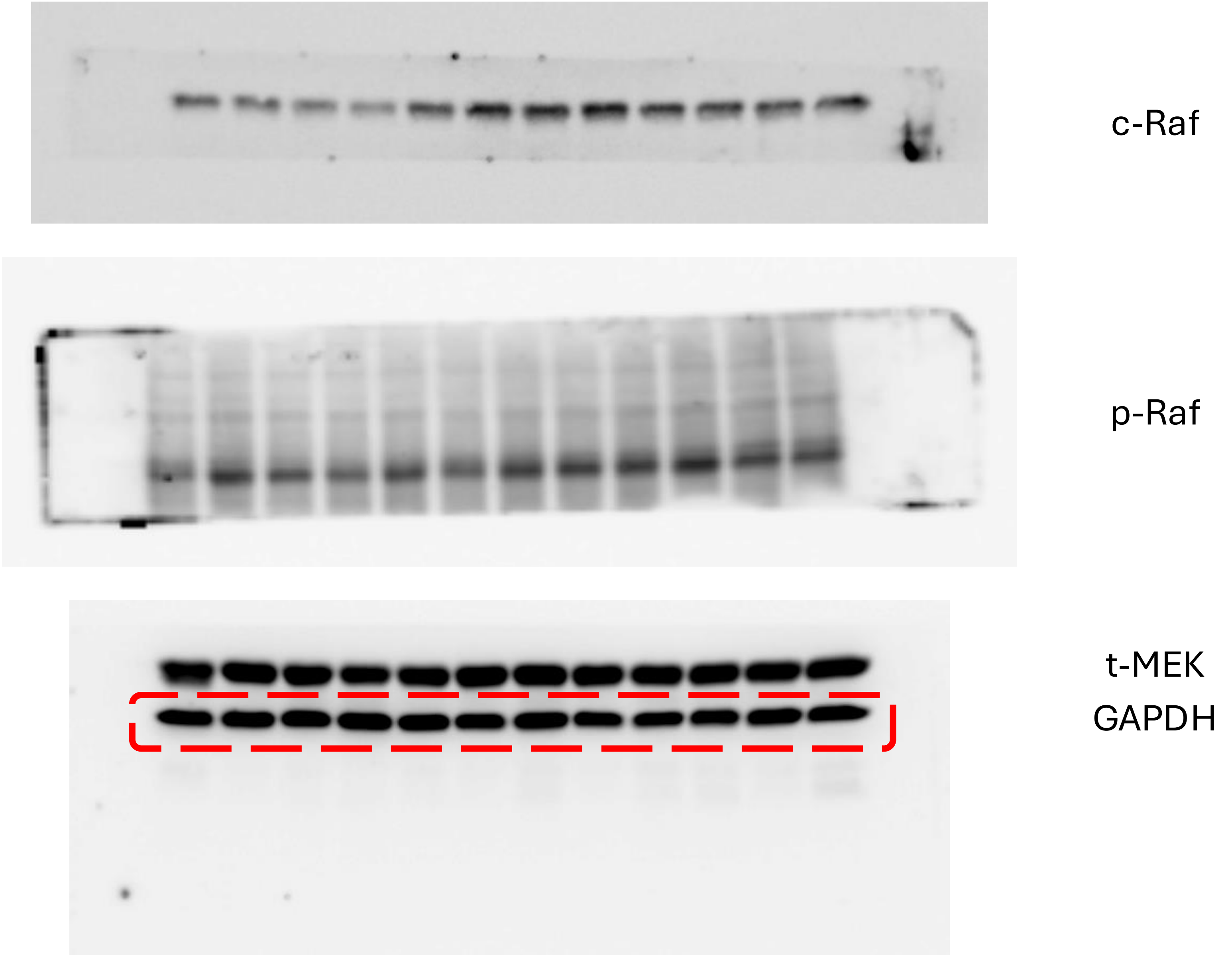

Note: A common loading control is shown for proteins analyzed on parallel gels loaded with the same lysates.
